# Enabling nondestructive observation of electrolyte composition in batteries with ultralow-field nuclear magnetic resonance

**DOI:** 10.1039/d5sc04419g

**Published:** 2026-01-29

**Authors:** Anne M. Fabricant, Román Picazo-Frutos, Florin Teleanu, Gregory J. Rees, Raphael Kircher, Mengjiang Lin, William Evans, Paul-Martin Luc, Robert A. House, Peter G. Bruce, Peter Krüger, John W. Blanchard, James Eills, Kirill F. Sheberstov, Rainer Körber, Dmitry Budker, Danila A. Barskiy, Alexej Jerschow

**Affiliations:** a Department of Biosignals, Physikalisch-Technische Bundesanstalt (PTB) Berlin Germany; b Institute of Physics, Johannes Gutenberg University of Mainz Mainz Germany budker@uni-mainz.de; c Helmholtz Institute Mainz Mainz Germany; d GSI Helmholtzzentrum für Schwerionenforschung Darmstadt Germany; e NVision Imaging Technologies GmbH Ulm Germany; f Extreme Light Infrastructure - Nuclear Physics, “Horia Hulubei” National Institute for Physics and Nuclear Engineering Bucharest Romania; g Interdisciplinary School of Doctoral Studies (ISDS), University of Bucharest Bucharest Romania; h Department of Chemistry, New York University New York NY USA alexej.jerschow@nyu.edu; i Department of Materials, University of Oxford Oxford UK; j The Faraday Institution Didcot UK; k Department of Chemistry, University of Oxford Oxford UK; l Quantum Technology Center and Institute for Research in Electronics & Applied Physics, University of Maryland, College Park Maryland USA; m Institute of Biological Information Processing (IBI-7), Forschungszentrum Jülich Jülich Germany; n Chimie Physique et Chimie du Vivant (CPCV, UMR 8228), Département de Chimie, École Normale Supérieure, PSL University, Sorbonne Université, CNRS Paris France; o Department of Physics, University of California Berkeley CA USA

## Abstract

Rechargeable batteries represent a key transformative technology for electric vehicles, portable electronics, and renewable energy. Yet, there are few nondestructive diagnostic techniques compatible with realistic commercial cell enclosures. Many battery failures result from the loss or chemical degradation of the electrolyte. In this work, we present measurements through battery enclosures that allow quantification of electrolyte amount and composition. The study employs instrumentation and techniques developed in the context of zero-to-ultralow-field nuclear magnetic resonance (ZULF NMR), with quantum magnetometers as the detection elements (atomic optically pumped magnetometers, OPMs, and superconducting quantum interference devices, SQUIDs, used in this work). In contrast to conventional NMR methodology, which suffers from skin-depth limitations, the reduced resonance frequencies in ZULF NMR make battery housing and electrodes transparent to the electromagnetic fields involved. As demonstrated here through simulation and experiment, both the solvent and lithium-salt components of the electrolyte (lithium hexafluorophosphate, LiPF_6_) signature can be quantified using our techniques. Further, we show that the ZULF-NMR apparatus and technique are compatible with measurements of pouch-cell batteries.

## Abstract 2

Ultralow-field nuclear magnetic resonance has the power to see chemistry through metallic enclosures—enabling nondestructive quantitative tracking of the state and composition of liquid-state electrolytes in batteries.

## Graphical abstract

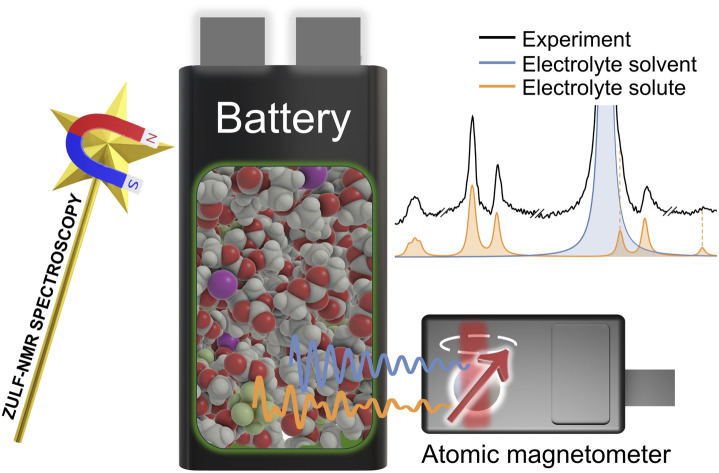

## Introduction

Rechargeable batteries, especially lithium-ion batteries, are already enabling great leaps in the electrification of transportation and the use of alternative energy sources. High-field nuclear magnetic resonance (NMR) of battery materials is a rich area of research, and many relevant electrochemical processes have been studied with this technique.^1–4^ One pain point of current technology, however, is the limited ability of analytical or diagnostic techniques to detect changes or defects within realistic battery cells (as opposed to purely research cells) in a nondestructive fashion. Recently, magnetic resonance imaging (MRI) was adapted to sense changes in the structure or magnetic susceptibility of battery materials, and thereby to provide a link between external measurements and internal processes in batteries.^5–8^ This type of indirect approach was further demonstrated with magnetometry, where atomic magnetometers were used to detect changes in the induced field as a function of applied background magnetic field^9^—specifically showing, for example, nonuniform lithium incorporation into the cathode. Further extensions of MRI- and magnetometry-based approaches to battery diagnostics include magnetic imaging of small (µA) currents either during charging/discharging or during resting periods,^9–11^ as well as the use of alternative detection media.^12^ Other types of sensors/modalities, such as magnetically induced tomography detected by nitrogen-vacancy (NV) centers in diamond^13^ allowed access to further observables for battery assessment. All these techniques provided the ability to probe either changes in solid components as a function of lithium (Li) incorporation, or changes in electrical current distributions through the measurement of magnetic fields around the batteries. The topic of nondestructive testing of batteries using magnetic techniques has been treated in some recent reviews.^14–16^

The electrolyte itself has so far not received much attention in the aforementioned approaches to nondestructive testing, nor was it generally possible to detect changes in electrolyte composition directly. The nature, distribution, and composition of the electrolyte are, however, critically important to the proper functioning of a cell. Changes such as leakage or electrolyte degradation due to aging processes are frequently the reason for battery failures.^17–19^

Typical battery electrolytes are mainly composed of a solvent—often a mixture of ethylene carbonate (EC) with dimethyl carbonate (DMC)—and the solute, a Li salt such as LiPF_6_.^20^ In this work, we aimed to study common battery-cell enclosures containing these chemicals, in order to access the characteristic spectroscopic signatures that would allow quantification of electrolyte amount and composition, including LiPF_6_ content. The measurement of electrolytes through aluminum (Al) enclosures is of particular interest, as Al is the typical housing for the commercial flat Li-ion pouch and prismatic cells widely used in electric vehicles, portable electronics, and renewable-energy storage.^21^

**Fig. 1:**
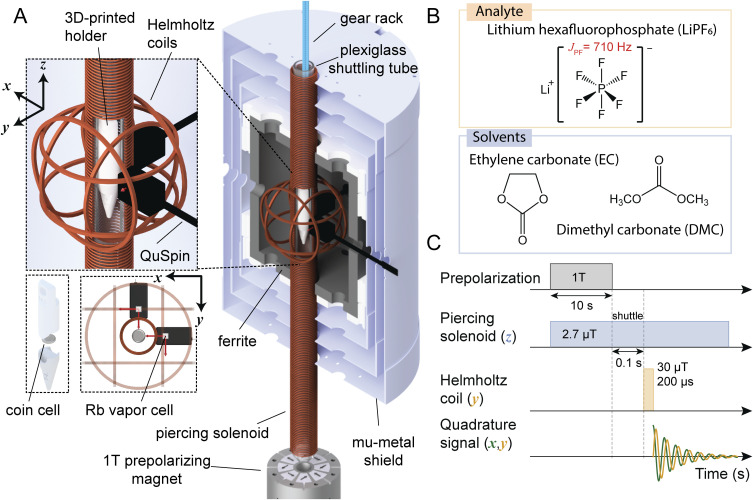
(A) Experimental ultralow-field NMR apparatus for mechanically shuttling thermally nuclear-spin-polarized samples to a hypogeomagnetic measurement region, where spin manipulation and subsequent measurement with atomic optically pumped magnetometers (OPMs) take place. The shuttling distance from the prepolarizing magnet to the measurement region is 36 cm. Cut-out shows the plastic holder containing a sample cell inside the piercing solenoid, where a tunable measurement field is used to produce magnetic resonances detected by a pair of OPMs (sensitive axes indicated by the four red arrows). The general shuttling setup was described previously,^26^ and further details of the specific operation of this apparatus are provided in Experimental. (B) Electrolyte (solvent and solute/analyte) chemical compositions studied in this work. (C) Measurement sequence for a single signal readout (“scan”) including prepolarization, shuttling, application of a 90° magnetic-field pulse to rotate magnetization into the detection plane, and detection of a decaying magnetic-dipole signal as the magnetization evolves freely in the applied solenoid field. Finally, the sample is shuttled back to the prepolarizing magnet for the next scan. Experiments typically consisted of many scans which were averaged to improve the measured signal-to-noise ratio (SNR) of electrolyte signals from sample cells containing less than 100 µL of electrolyte.

One option for obtaining spectroscopic electrolyte signatures from the inside of a cell is to examine it with NMR. At the frequencies commonly employed in NMR spectroscopy (hundreds of MHz), however, the skin depth of electromagnetic radiation in metal is only on the order 10 µm (Fig. S1), which prevents fields from penetrating the cell during nuclear-spin excitation and detection. Although demonstrations at such high frequencies exist, quantification and reproducibility are challenging due to field-shaping effects and tuning variabilities.^22,23^ Because skin depth scales inversely with the square root of frequency (Fig. S1), even low-field benchtop instruments (based on permanent magnets, with proton precession frequencies on the order 10 MHz) still only enable penetration of tens of µm of metal at best. For this reason, and due to sample-size limitations, battery testing with benchtop NMR is typically limited to studies of research pouch cells which fit into an NMR tube, or to inline studies of redox flow cells.^24^ By contrast, in zero-to-ultralow-field (ZULF) NMR experiments (Fig. 1), the resonance frequencies of nuclear-spin samples can span the range from Hz to kHz and are tunable through the application of a background field. Spin excitation is typically performed using pulses of static magnetic fields, and metals are therefore essentially transparent to the applied and measured electromagnetic fields.^25^

In traditional NMR spectroscopy, internal couplings and especially *J* couplings—indirect spin–spin couplings mediated by the electrons shared in chemical bonds—are much smaller than the Zeeman interaction. In the ZULF-NMR regime, the situation is opposite: the Zeeman interaction is much weaker than the *J*-coupling interaction, such that Zeeman coupling represents a perturbation to the *J*-coupling Hamiltonian. Thus, the molecular information is encoded in so-called *J* spectra,^27,28^ rather than in chemical-shift values. These spectra, which arise from angular-momentum selection rules (see SI), can be used to obtain molecular fingerprints of studied samples. ZULF-NMR spectra do not suffer from susceptibility-induced line broadening even in materials with complex internal structure, and consequently display narrower spectral lines compared to higher-field measurements.^29–31^

At the relatively low frequencies of signals in ZULF-NMR spectroscopy, inductive detection is largely ineffective due to decreased sensitivity, so detection is typically performed with quantum sensors—either superconducting-quantum-interference-device (SQUID) magnetometers^32^ or (noncryogenic) atomic magnetometers, also called optically pumped magnetometers (OPMs).^33^ Both SQUIDs and the most sensitive OPMs are generally operated in a near-zero-field environment, where Earth's magnetic field is screened by means of magnetic shielding. Furthermore, to boost signals, nonequilibrium spin polarization of samples is created either by prepolarizing them in a stronger magnetic field (using a permanent magnet or electromagnet) or by employing hyperpolarization techniques.^34,35^ With these implementations, ZULF NMR has been successfully applied to studies of fundamental physics,^36,37^ chemical fingerprinting of biological samples and metabolism using *J* spectroscopy,^30,31,38^ as well as relaxometry at hypogeomagnetic fields.^26,39^ Battery diagnostics represent a new direction in the application of ZULF-NMR instrumentation.

To demonstrate the sensitivity of our method to the smallest possible realistic volume of battery electrolyte inside battery housing, experiments are presented on ≈1 mm-thick Al coin-cell enclosures with a sealed form factor and containing less than 100 µL of electrolyte, using an OPM-based setup (see Experimental). We find that ZULF-NMR spectroscopy allows the detection and assignment of electrolyte signals such that molar concentrations, as well as changes in composition and potentially degradation, can be quantified. The spectral signature and measured electrolyte composition are benchmarked against *in vitro* calibration data obtained with the OPM-based ZULF setup, a SQUID-based ZULF setup, and a commercial benchtop device (Experimental). In addition, we show first ZULF-NMR recordings of pouch-cell batteries, using both OPM and SQUID detection. Our work establishes the ability of ultralow-field NMR to noninvasively and reliably detect electrolyte inside batteries of various materials and geometries.

## Results and discussion

In this work, we primarily employ a ZULF-NMR setup based on thermal prepolarization, mechanical shuttling between the prepolarization and detection regions, and room-temperature, quadrature detection using commercially available atomic OPMs.^40^ The experimental apparatus and measurement scheme were described previously.^26^Fig. 1 depicts the apparatus as used in the experiments presented here (further details are provided in Experimental). Photos of the measured sample cells, along with the cell holder, are shown in Fig. S2.

**Fig. 2:**
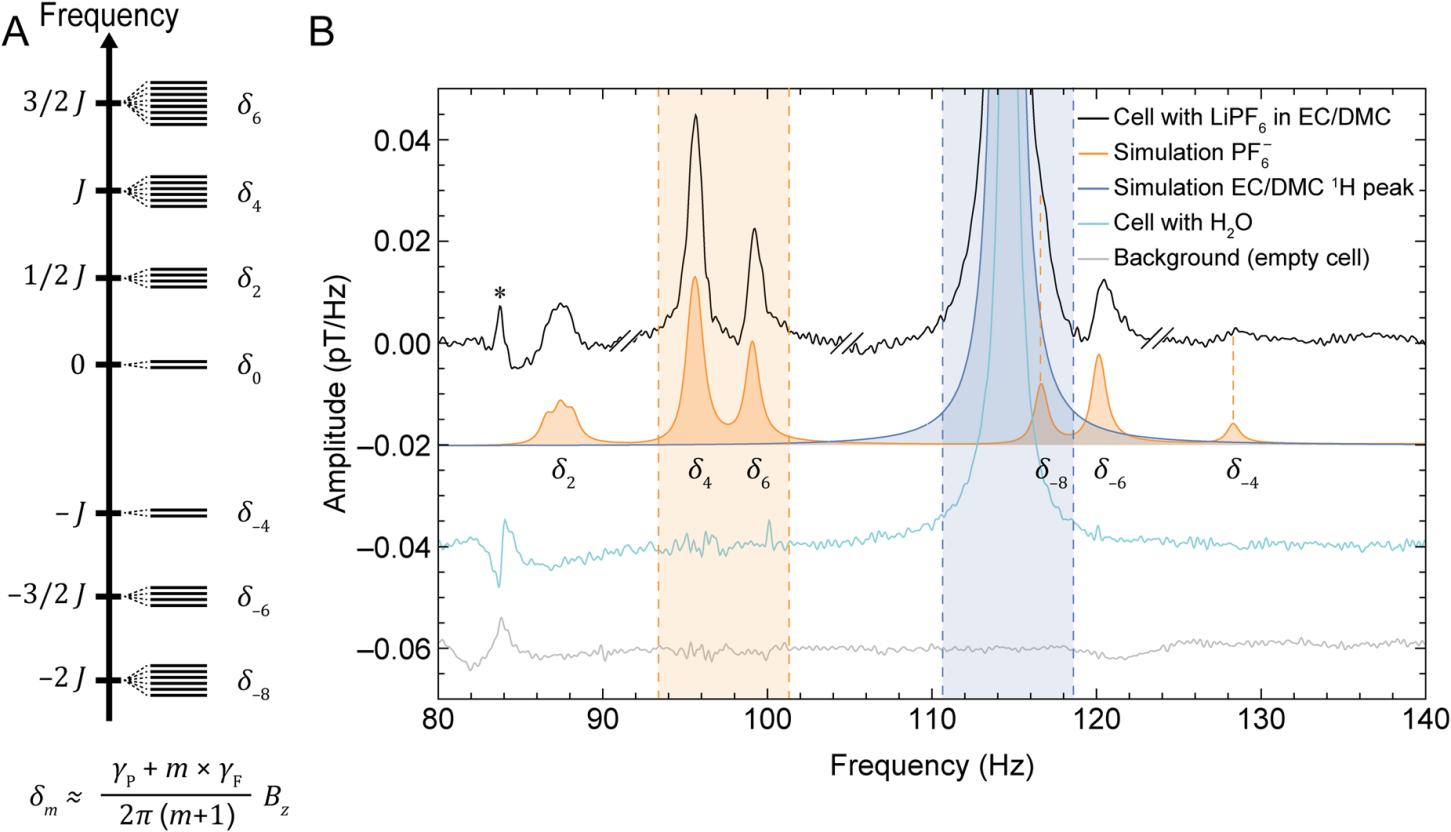
(A) Energy-level diagram showing manifolds containing eigenstates of the *J*-coupling Hamiltonian for the PF_6_^−^ spin system (see SI for further details). Application of a background magnetic field *B*_*z*_ in the solenoid (Fig. 1) lifts the degeneracy of the eigenstates within each manifold, splitting the energy levels as indicated schematically. Each *δ*_*m*_, where *m* is an integer, refers to the transition frequency between energy levels of the same manifold. Here, *J* = *J*_PF_ ≈ 710 Hz and the Zeeman splitting is approximately linear in the ultralow-field regime; *γ*_P_ and *γ*_F_ are the gyromagnetic ratios of phosphorus and fluorine, respectively. (B) Characteristic measured and simulated NMR signals at a 2.7 µT background magnetic field, using the setup and protocol depicted in Fig. 1. The graph displays the recorded electrolyte signature from a sample cell (black), a background noise measurement of an empty cell (gray), simulation of the PF_6_^−^ spin system (orange), simulation of the solvent proton signal (blue), and a recorded spectrum from an identical cell containing deionized water for calibration (cyan). The near-zero-frequency (nZF) peaks corresponding to the *δ*_*m*_ transitions are labeled beneath the simulated spectrum. Experimental spectra are obtained from averages of 10 000 scans. The shaded areas indicate the frequency ranges used for calculation of concentrations, as described in the main text. Electrolyte experimental data (black) was phased using the relative zeroth-order phases −30°, 150°, 0°, and −90° in four different spectral regions, respectively (see Experimental and SI); double dashes delimitate these phased regions. The spectra are offset for visual clarity, and vertical orange dashed lines provide an aid to the eye for the measured PF_6_^−^ peaks with lower SNR. The SNR of the PF_6_^−^ peaks are 13, 74, 37, 20, and 5 for the peaks at approximately 87, 96, 99, 115, 120, and 128 Hz, respectively—calculated as the maximum signal amplitude divided by the standard deviation of a neighboring noise region from 132 to 140 Hz. The linewidths (full width at half maximum, FWHM) of these peaks range from 1 to 1.5 Hz.

The electrolytes selected for the main study were composed of different amounts of LiPF_6_ dissolved in a 50 : 50 (v/v) mixture of EC/DMC (Fig. 1B). In ZULF-NMR spectra, one therefore expects to observe a lower-frequency family of signals—depending on the background field and associated Larmor frequencies—which arise from Li^+^, PF_6_^−^, and the EC/DMC solvent protons (henceforth referred to as the near-zero-frequency peaks, nZF peaks). Specifically, we are dealing with ^7^Li, coupled ^31^P and ^19^F spins, and ^1^H. In the case of the PF_6_^−^ system, the Zeeman interaction with the detection field (produced by the piercing solenoid, Fig. 1) lifts the degeneracy inside manifolds of total spin angular momentum *F*, leading to transitions between states inside the same manifold observed as nZF peaks (SI text and Fig. S3 and S4). In addition, the PF_6_^−^ unit gives rise to transitions at higher frequencies of 3/2 *J*_PF_, 5/2 *J*_PF_, and 7/2 *J*_PF_, where *J*_PF_ ≈ 710 Hz (exact value depending on solvent composition) is the *J*-coupling constant between ^31^P and ^19^F nuclei^28^—these signals are referred to as *J* peaks in the following). See Fig. 2A for the energy-level diagram of this system, depicted in more detail in Fig. S3.

For the implementation of our method, we chose to focus on the nZF manifold for two reasons: (1) the *J* peaks are on order 100 times weaker than the nZF peaks for PF_6_^−^, which would complicate the measurement of smaller sample volumes within a reasonable amount of time; (2) the higher frequencies of the *J* peaks fall outside the sensitive bandwidth of the OPMs used—signals are detectable up to 500 Hz, with flat sensor response in a 100 Hz band.^40^

Fig. 2 shows a characteristic electrolyte spectrum measured from a sample cell of coin-cell geometry (Fig. S2), as described in Experimental, at a detection field of 2.7 µT. Although nZF peaks could in principle be measured at arbitrarily low fields, practical considerations motivated a choice of field in the microtesla range, corresponding to a ^1^H Larmor frequency of approximately 115 Hz; all PF_6_^−^ and EC/DMC signals appear within the spectral range 86–130 Hz. This approach allowed us to move the signals of interest out of a lower-frequency region where significant noise was observed due to shuttling of the conductive aluminum housing (Fig. S5). The complexity of the PF_6_^−^ nZF manifold is reproduced by simulations using the Spinach package in MATLAB^41^ and stems from lifting the degeneracy inside spin manifolds due to the Zeeman perturbation of ZULF eigenstates,^42,43^ as illustrated in Fig. 2. A simulation of the EC/DMC proton signal is also included, as well the measured water-proton signal from a calibration cell filled with deionized water. Finally, the background signal from an empty cell is displayed to identify artifacts not arising from the spin sample, such as the noise peak at 84 Hz. The sample volume was only about 80 µL, and it is thus quite promising that electrolyte NMR signals can be obtained from such a small volume using quantum magnetometers. For completeness, we note that we were also able to identify the Li^+^ signal at lower frequency, here around 45 Hz (Fig. S6C). For subsequent analysis, however, the signals of PF_6_^−^ and EC/DMC were used, since they had larger SNR and were frequency-separated from spectral noise features.

In order to consistently extract electrolyte concentrations from all recorded experimental spectra, solute and solvent signals were integrated over the shaded regions indicated in Fig. 2—including the two largest PF_6_^−^ peaks in the spectral window 93.38–101.32 Hz, and the solvent proton peak in the spectral window 110.65–118.59 Hz. Although the latter integration region also contains a smaller PF_6_^−^ signal around 116 Hz, this contribution is negligible compared to the much larger (by two orders of magnitude) proton signal.

**Fig. 3:**
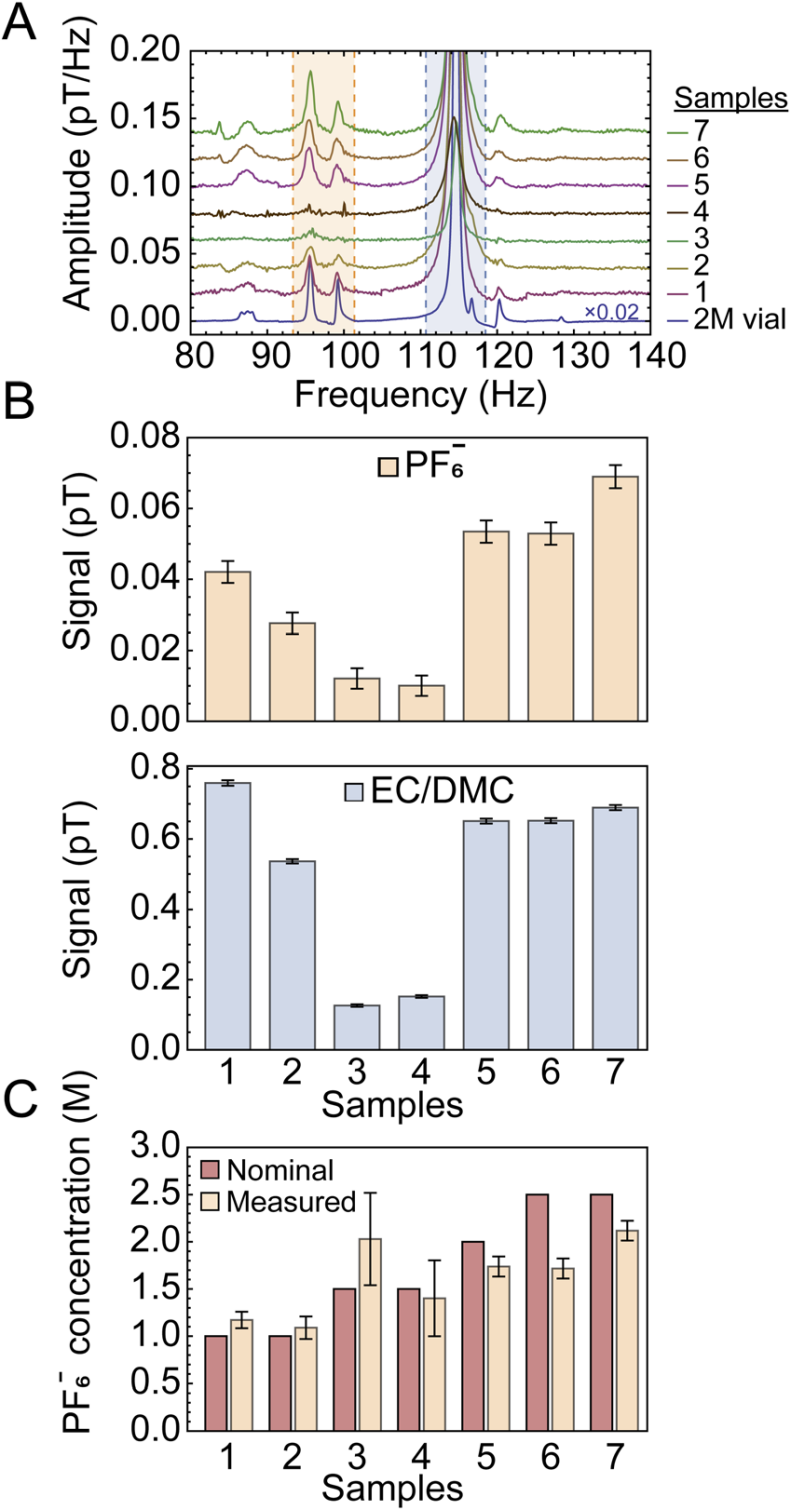
(A) Stacked plots showing the measured electrolyte signals at a 2.7 µT background field from a series of sample cells filled with electrolyte of different nominal (prepared) LiPF_6_ concentrations. All spectra were obtained with the setup and protocol in Fig. 1 from averages of 10 000 scans, apart from sample 1 and the calibration vial for which 8913 and 256 scans were collected, respectively; the large number of scans was selected to improve SNR of measured signals from the cells, as well as to suppress the power-line harmonic at 100 Hz. (B) Quantification of signals obtained from integration of the shaded areas indicated in (A) for the solute and solvent peaks (top and bottom panels, respectively). Error bars correspond to the standard errors obtained in Fig. 4 and their values as a fraction of the signal scale inversely with SNR, as explained in the text. (C) LiPF_6_ concentrations obtained from the measurements in (B) and propagation of errors, according to the procedure outlined in the text.

Fig. 3(A) presents the spectra for a series of samples prepared with different electrolyte concentrations (1.0–2.5 M) and approximately the same total liquid content. The bottom trace in Fig. 3A was acquired using a reference vial of known electrolyte concentration (off-the-shelf LP30 solution, see Experimental). In Fig. 3B, the solute and solvent signals are compared for each sample based on the integrals extracted from the indicated shaded spectral regions. The ratio of integrated solute and solvent signals was compared to the signals from the calibration-vial data (Fig. 3A and S6A) in order to obtain normalized concentration values. Fig. 3C shows the calculated PF_6_^−^ concentration for all cells, normalized to the 2 M calibration sample, following the relation1
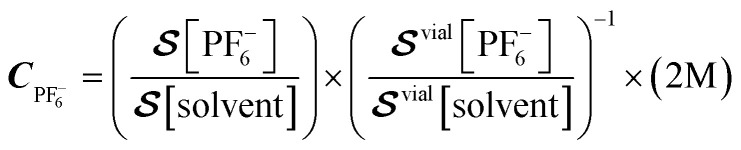
Here, 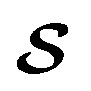 is the measured signal (integrated area of the peak, Fig. 3B). We chose this calibration approach in part because the total liquid amount in each cell varied due to the production method (in the process of sealing the cells, some spillage was inevitable). Therefore, comparing signal ratios between the samples and reference vial data allowed us to remove uncertainty arising from different liquid amounts or potential leakage. The signal from the vial is much larger than that from the sample cells (Fig. 3A), due to both an increased sample volume (the vial contained 1.5 mL of electrolyte while the cells typically contained 80 µL) and a more efficient geometry (the sensor arrangement depicted in Fig. 1) is more suitable for the approximately cylindrical geometry of the vial, rather than the disc-like coin cells). These factors, as well as possible reduction of magnetization due to fields induced by shuttling conductive material (see SI), do not affect the relative quantification method of eqn (1). For samples 3 and 4, the SNR of electrolyte signals, barely visible in Fig. 3A, are relatively low, and hence the calculated concentrations have larger error bars in Fig. 3C.

**Fig. 4:**
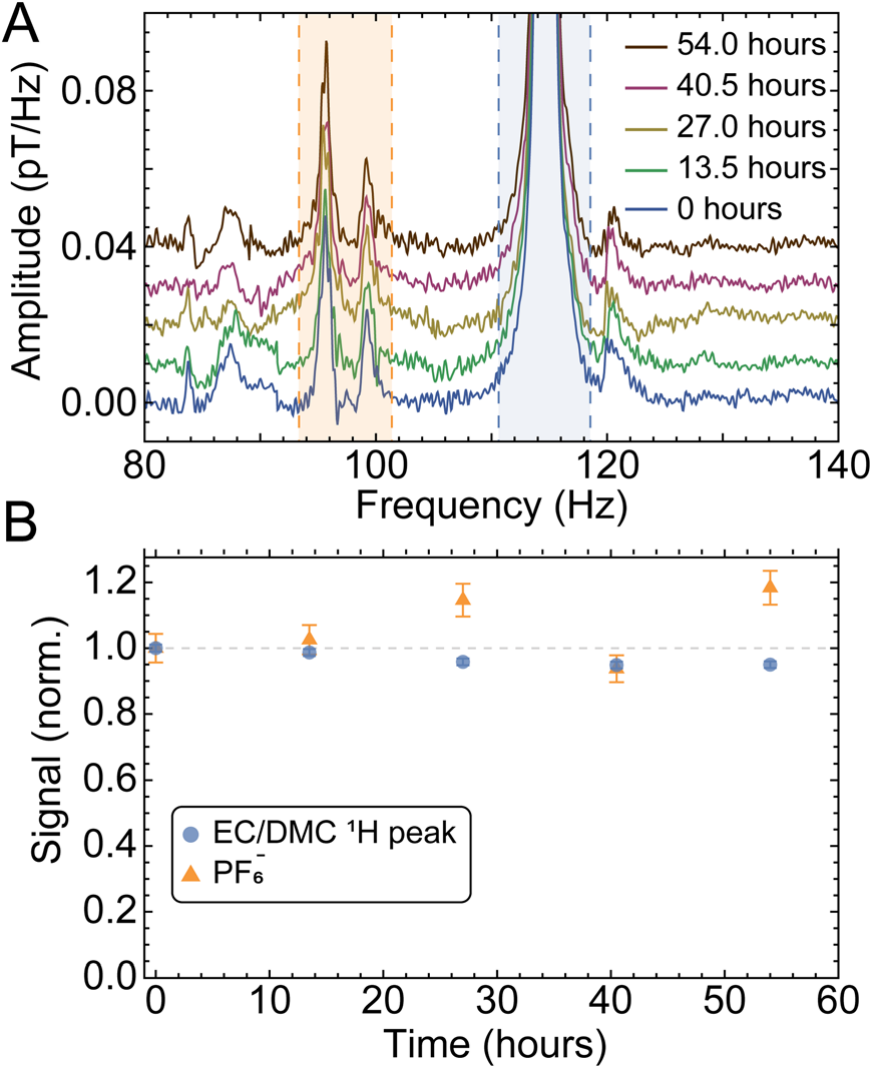
Monitoring of the electrolyte peaks as a function of time, using five partitions or batches of 2000 scans (13.5 h each) from the cell with a nominal 2.5 M LiPF_6_ concentration (sample 7 in Fig. 3). (A) Measured spectra, and (B) solute and solvent integrals normalized to the first data point. Error bars are calculated as standard error of the partitioned integrated signals (shaded regions). The error on the proton signal is smaller than that on the PF_6_^−^ signal (relative errors of 4.4% and 1.1% for solute and solvent signals, respectively), as expected due to the smaller SNR of the latter. The electrolyte signals from this sample cell were found to be relatively stable not only over the course of the three-day measurement cycle (with minor fluctuations), but also in a second measurement taken two months later (Fig. S7).

To demonstrate the robustness of our setup and the reproducibility of measurements, we analyzed partitions of data from the same (largest-SNR) sample at different time intervals under identical experimental conditions, as displayed in Fig. 4. The standard errors of solute and solvent signals extracted from this data set were used to calculate the error bars displayed in Fig. 3. In this analysis, uncertainty on measured signals (peak integrals) is assumed to scale inversely with SNR, such that higher SNR corresponds to a smaller error bar (see SI for further details). These error bars account for both statistical uncertainty as well as possible systematic uncertainty over the course of the measurement cycle.

As is evident from Fig. 3C comparing the measured LiPF_6_ concentrations to the nominal (prepared) concentrations, for the majority of samples, the measured concentrations agree with the nominal values to within 10%. Furthermore, the relative stability or loss/leakage of electrolyte signals could be tracked through time-separated measurement of the same cells (Fig. S7 and S8, respectively). Only samples 6 and 7 display larger deviations between nominal and measured concentrations—this may be due to production systematics or the fact that signal size and linewidth can affect the percentage of peak area contained within the integration bounds.

To test compatibility of the experimental setup and protocol not only with Al housing but also with all other components of a realistic working battery, additional coin cells of standard geometry were tested—without electrolyte but containing a copper current collector, a lithium anode, a glass-fiber separator, and a lithium-nickel-manganese-cobalt-oxide (NMC811) cathode. Although the inclusion of copper material can increase the amount of background noise attributed to shuttling-induced eddy currents (Fig. S9), this was not expected to impede measurement or characterization of electrolyte content. The baseline noise occurs at lower frequencies and resonance frequencies in the setup can be shifted out of this range through tuning of the measurement field (Fig. 1), or one can drop more initial points of the measured time-domain signal to suppress the noise (Fig. S9).

**Fig. 5:**
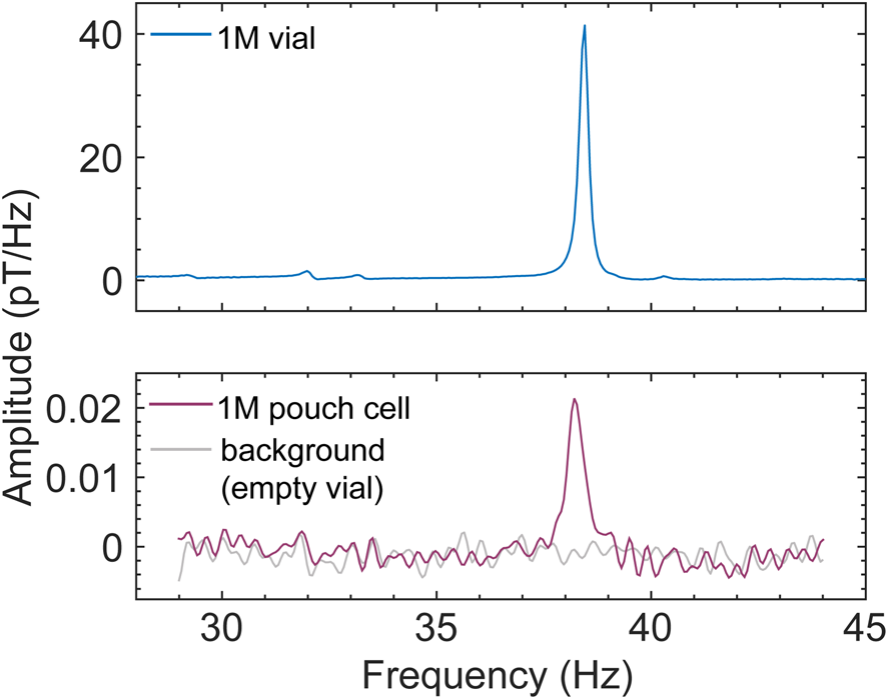
Results from functional pouch-cell batteries demonstrate that they can be measured with our shuttling apparatus (Fig. 1 and S10). The spectrometer could be operated at an arbitrarily low background field—here 0.9 µT, corresponding to a proton resonance frequency of 38 Hz—due to the reduced shuttling-induced baseline noise from the laminate pouch-cell casing. Plots compare recorded spectra from a 1.5 mL calibration sample of industry-standard 1 M LiPF6 in 50 : 50 EC/DMC (top panel, 256 scans), a miniature pouch cell filled with 38 µL of the same electrolyte (bottom panel, darker trace, 10 000 scans), and an empty sample vial showing the noise floor of the spectrometer (bottom panel, lighter trace, 10 000 scans). See also Fig. S11D.

With these encouraging results, we turned our attention to functional pouch-cell batteries, which are most relevant for industrial applications. It was necessary to manufacture these in-house, as commercially available miniaturized pouch cells which could fit into the bore of our prepolarizing magnet and solenoid in the shuttling setup (Fig. 1) typically contain polymer rather than liquid electrolyte (this reduces the cell weight for wearable electronics, for example). Our aim was rather to test a scaled-down version of standard liquid-electrolyte cells as a proof of principle. To this end, pouch cells with a 1 cm^2^ active area were produced from commercial components, with a lithium-cobalt-oxide (LCO) film cathode, a graphite anode, and battery-grade 1 M LiPF_6_ in EC/DMC (LP30) as the electrolyte (Experimental). Due to the miniscule electrolyte volume and the flat geometry of the pouch cell (Fig. S10) which did not fill the cylindrical sensitive region of the spectrometer, as well as possible electrolyte absorption into the separator or around the paramagnetic LiCoO_2_ cathode, only the proton solvent signal is visible above the noise. The largest PF_6_^−^ peak appears in a calibration spectrum of the same electrolyte at 32 Hz, with an amplitude a few percent that of the proton peak at 38 Hz, as expected for the 1 M concentration. Despite this, the solvent proton signal from the cell was clearly visible at the expected frequency (Fig. 5)—already sufficient to characterize the presence (or absence) and potential leakage of liquid electrolyte. Currently, the state of the art for diagnosing electrolyte leakage from batteries is to detect evaporated solvent or associated volatile organic compounds (VOCs) outside the battery using gas or optical sensors,^16,44,45^ after significant dangerous leakage with associated fire risk may have already occurred.^46,47^

As a technical note, we have found that higher conductivity of metals is associated with larger shuttling-induced eddy currents (see Fig. S5 and S9)—which is likely why the less pure commercial-grade Al pouch-cell foil does not suffer from this issue. Nonmagnetic (such as Al) battery enclosures are preferred in our OPM-based setup, because the particular sensors used for detection can only operate in background fields less than 100 nT.^40^ This is not a fundamental limitation, however, since a variety of OPM sensor options exist for operation at elevated fields and even in unshielded environments—as described in recent reviews and references therein.^48–50^ Furthermore, the OPMs used in this work have been shown to be compatible with common commercially available Li-ion pouch cells.^9^ Of course, understanding the material properties of various measured battery cells is critical to the design of optimized NMR experiments.

**Fig. 6:**
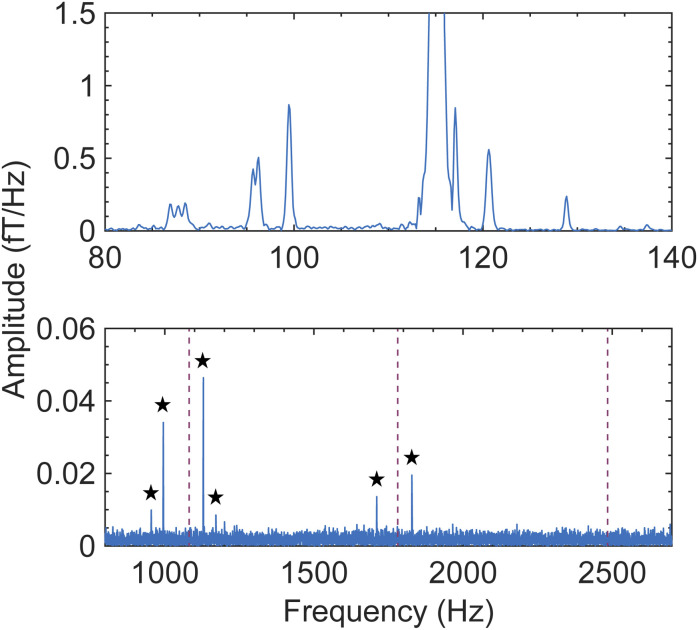
Ultralow-field NMR magnitude spectrum of a 20 mL solution of 1 M LiPF_6_ in EC/EMC, detected using a second ZULF setup with a SQUID gradiometer as the detection element (see Experimental and Fig. S11A). The sample was polarized *in situ* for 5 s by an electromagnet at 6 mT, which was then switched off for subsequent SQUID detection in a background field of 2.7 µT. Polarization and detection were performed along parallel axes; data acquisition lasted 5 s at 7.5 kHz sampling rate and 5000 averages were collected. Top panel: SQUID-detected nZF peaks of PF_6_^−^ and proton solvent peak at 115 Hz, matching the signature which was detected using the OPM-based shuttling setup (Fig. 1–4). The SQUID spectrum, which was collected inside a magnetically shielded room, displays narrower spectral lines likely due to improved field homogeneity and/or relaxation differences. Because individual ZULF peaks can also relax at different rates,^51^ the relative amplitudes of the various PF_6_^−^ spectral lines differ slightly from those observed with the shuttling apparatus, attributed to the distinct spin-preparation protocols and timings of the two experiments. However, the pattern and positions of spectral lines are the same in both cases. Bottom panel: *J* spectrum of PF_6_^−^ collected simultaneously with the nZF spectrum, making use of the larger bandwidth of SQUID sensors. Splitting of the *J* peaks around of 3/2 *J*_PF_, 5/2 *J*_PF_, and 7/2 *J*_PF_ (red dashed lines) is expected (Fig. S3); here the four largest peaks (marked with black stars) are visible above the noise. The much smaller amplitudes of *J* peaks compared to nZF peaks in the recorded ULF spectrum validates the choice to focus on the latter in this work.

As a next experimental step, we sought to benchmark the observed ZULF-NMR LiPF_6_ spectrum using a second independent apparatus, in order to check reproducibility. For this purpose, a completely different setup was employed, featuring *in situ* thermal polarization at a lower field (6 mT) rather than shuttling and a SQUID gradiometer rather than OPM gradiometers as the detection elements—see Experimental for a detailed description. Strikingly, even with this independent apparatus and protocol, as well as a different solvent composition (ethylene carbonate, EC, with ethyl methyl carbonate, EMC) the same PF_6_^−^ ULF signature is observed at 2.7 µT (Fig. 6, top panel, and Fig. S11A). Furthermore, the broader (2.5 MHz) bandwidth of the SQUID system allows for simultaneous detection of the PF_6_^−^*J* spectrum (Fig. 6, bottom panel), whose spectral pattern matches simulation (Fig. S3). After collection of this calibration data from a reference vial, larger pouch cells containing the same electrolyte mixture—1 M LiPF_6_ in 30 : 70 (% w/w) EC/EMC—were measured with the SQUID apparatus. Despite the fact that each cell contained a liquid electrolyte layer of only sub-mm thickness (Experimental), the solvent proton signal was clearly visible, and stacks of cells could be measured simultaneously despite increased Johnson noise from the metallic cell components (Fig. S11B–D). Thus, both OPM- and SQUID-based ZULF setups were demonstrated to be compatible with realistic commercial battery materials and geometries.

**Fig. 7:**
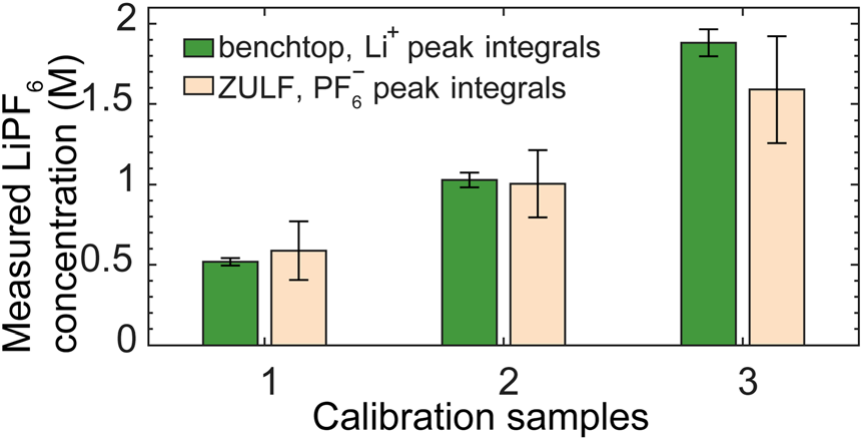
Quantification comparison of electrolyte calibration samples recorded using both the OPM-based ZULF apparatus (Fig. 1) at 2.7 µT and a commercial 1.4 T benchtop spectrometer (Experimental). Solutions of LiPF_6_ in EC/DMC were prepared in the ZULF-NMR lab from individual compounds, at three nominal concentrations {0.5, 1, 2} M for comparative measurements. Integrating over the largest LiPF_6_ manifold in each spectrum—the Li^+^ peak at 0.5 ppm in the benchtop data, integrated over +4 to −4 ppm, and the PF_6_^−^ nZF peaks in the ZULF data, integrated over the bounds shown in Fig. 2—allowed for extraction of relative concentrations. Bars (green: benchtop; beige: ZULF) and error bars represent the average and standard deviation of normalized concentrations for the three possible normalizations, with smaller error bars on the benchtop data corresponding to larger SNR of spectra. The two sets of results agree to within experimental uncertainty, validating the integration-based quantification method. The lower amplitude of the third sample with respect to the nominal 2 M value may reflect incomplete dissolution of the LiPF_6_ salt in the homemade solution, motivating our use of an off-the-shelf mixture for absolute quantification in Fig. 3.

In addition, for further validation of the quantification procedure and spectral reliability, we prepared LiPF_6_ calibration samples of different nominal concentrations for comparative measurement using both the OPM-based ZULF spectrometer and a 1.4 T benchtop spectrometer (Experimental). By integrating over the largest spectral lines in each case—PF_6_^−^ for the ZULF recording and Li^+^ for the benchtop recording—we compared relative measured concentrations as shown in Fig. 7. We found that the ULF and benchtop results agree to within the experimental uncertainty, despite having been obtained in separate field regimes and from different nuclear species of the analyte. Finally, we performed another verification that the PF_6_^−^ ULF signature was stable even under change of electrolyte composition (from 50 : 50 (v/v) EC/DMC to 80 : 10 : 10 (v/v/v) EC/DMC/DEC, where DEC is diethyl carbonate), as shown in Fig. S6D.

## Experimental

Additional experimental, as well as theoretical, details are available in the SI.

### Materials and methods

The sample cells forming the primary data set of this article were manufactured in February 2024 and measured March–April 2024; miniature pouch cells were manufactured in October 2024 and measured November–December 2024. SQUID and benchtop benchmarking tests were conducted September–November 2025. Additional experimental results, calibration data, simulations, and photographs are available in the SI.

### Instrumentation and data collection

The shuttling setup (Fig. 1A) and SNR-enhancing “gradiometric quadrature” OPM detection method were described in detail in previous work,^26^ where device calibration and applications of proton relaxometry were discussed. In this work, the apparatus was primarily operated at a constant background field of 2.7 µT along 
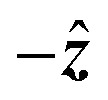
 inside the double-layer piercing solenoid, corresponding to an applied current of 600 µA and a proton precession frequency of 114.68 Hz (Fig. 2). Prior to measurement, each sample cell was enclosed in a 3D-printed PLA holder affixed to the plastic gear rack and positioned inside the 1 T permanent magnet (Halbach array). A single measurement cycle (Fig. 1C) consisted of: (1) 10 s nuclear-spin polarization in the magnet, (2) 100 ms shuttling 36 cm into the detection region at the center of the magnetic shield (Twinleaf MS1-LF), (3) application of a 30 µT π/2 magnetic-field pulse along −*ŷ* to rotate magnetization into the *x*–*y* detection plane (Fig. S12), (4) at least 5 s four-channel acquisition of the oscillation/decaying spin signal by two dual-axis QuSpin Zero-Field Magnetometers (QZFM Gen-2) during magnetization precession in the background field, and (5) return of the sample to the starting position inside the magnet. One sensor was pointing along the *x*-direction and the other along the *y*-direction, to enable quadrature detection.^26^ The cylindrical cell holder (Fig. S2) has outer diameter 14 mm and inner diameter 10 mm. In a typical experiment, 10 000 scans were averaged, a 200 µs pulse was applied to both proton and PF_6_^−^ spin systems (Fig. S12), and the duty cycle was approximately 20 s with several seconds of rest between scans.

The ultrasensitive SQUID system consisted of a first-order axial gradiometer inductively coupled to a current-sensing SQUID for data acquisition along the *z*-axis (Fig. S11A). The sensor probe contained a gradiometric pick-up coil with diameter 45 mm and baseline 120 mm and was operated in the liquid-helium dewar LINOD2 ^52^ which features negligible magnetic noise, reaching a white-noise level of ≈200 aT/√Hz (Fig. S11C). As pictured in Fig. S11A, this SQUID system was located inside a three-axis ULF magnetic-resonance-imaging (MRI) coil system, with the sample to be measured placed under the dewar at the center of the coil frame. Offset distance from the pick-up coil to the sample was 12.9 mm, defined by the thickness of the dewar wall and insulation. The whole setup was operated in the moderately magnetically shielded room “ZUSE MSR” (two layers of mu-metal and one eddy-current shield), achieving residual fields of <1.5 nT after degaussing.^53^ Measurements were conducted *in situ* using two sets of coils (Fig. S11A): samples were prepared for 5 s in a 6 mT prepolarizing field (coil calibration factor 48.3 µT/A) which was then rapidly switched off within a few ms for immediate detection in the *x* background field (coil calibration factor 295 µT/A). Depending on the experiment, the prepolarizing field was either directed along *ŷ* (for battery measurements, Fig. S11) or along 
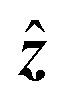
 (for electrolyte calibration measurements and collection of *J* spectra, Fig. 6. The detection field was tunable from nominally zero up to ≈40 µT.

For the collection of benchtop-NMR data (Fig. 7 and S13B), samples were prepared and sealed in a glovebox under inert atmosphere (N_2_) in 5 mm NMR tubes. Benchtop measurements on ^19^F and ^7^Li nuclei were carried out with an instrument from Magritek (Spinsolve 60 Multi X, 1.4 Tesla). Parameters for ^19^F measurements (57.7 MHz, *cf.* Fig. S13B) were as follows: acquisition time of 3.3 s, relaxation delay of 10 s, flip angle of 90°, and 32 scans. Parameters for ^7^Li (23.8 MHz) measurements were as follows: acquisition time of 6.5 s, relaxation delay of 15 s, flip angle of 90°, and eight scans. The ^7^Li benchtop spectra were processed with the software MesReNova and used for reference quantification of various concentrations of LiPF_6_ in EC/DMC, *cf.*Fig. 7.

### Battery and calibration samples

The AG7 coin-cell cases were constructed from ultrapure aluminum metal (99.9%) in a battery-production facility. Lithium hexafluorophosphate (LiPF_6_ (s)) salt was dissolved into a 50 : 50 (v/v) mixture of ethylene carbonate (EC: (CH_2_O)_2_CO (s)) and dimethyl carbonate (DMC: OC(OCH_3_)_2_ (l)), to form 0.5, 1 (LP30), 1.5, 2, and 2.5 M salt-concentration electrolytes. A total of ≈80 µL of the various-concentration electrolytes was then pipetted into each of the coin-cell cases. The coin cells were sealed using a homemade plastic insert, to prevent magnetic impurities from the coin-cell crimper which might generate excessive static magnetization. All samples were stored, handled, and processed in an argon atmosphere (<1 ppm H_2_O, <1 ppm O_2_). Each sealed cell had an outer diameter of 9.4 mm and height 2.6 mm (Fig. S2); the average thickness of the side wall through which electrolyte signals were measured was around 1 mm. The interior dimensions of the cells were approximately 7.3 mm (diameter) by 2 mm (height).

The miniature pouch cells (Fig. 5 and S10) were assembled with casted LiCoO_2_ as the cathode and graphite as the anode, with the pouch consisting of a mixture of 20 µm-thick Al and glue. LiPF_6_ salt was dissolved into a 50 : 50 (v/v) mixture of EC and DMC to form 1 M salt-concentration electrolyte. A total of 30–180 µL was then pipetted into a 9.5 mm^2^ region within each of the pouch cells. The cells were vacuum-sealed, stored, handled, and processed under an argon atmosphere (<1 ppm H_2_O, <1 ppm O_2_).

For the SQUID measurements of larger-format pouch cells (Fig. S11B–D), single-layer cells were assembled using graphite anodes and lithium-iron-phospate (LFP) cathodes cut from roll-stock electrode foils. The electrodes were cut to a total length of 180 mm, with active coating lengths of 150 mm (anode) and 145 mm (cathode). Anodes were trimmed to a width of 60 mm and cathodes to 55 mm, resulting in active areas of 90 cm^2^ and 79.75 cm^2^, respectively. The graphite anode consisted of 95 wt% active material with a total thickness of 115 µm (8 µm Cu substrate), 24.4% porosity, an areal capacity of 2.65 mAh cm^−2^, and a coating load of 80 g m^−2^ per side. The LFP cathode comprised 93 wt% active material with a total thickness of 178 µm (12 µm Al substrate), 38.5% porosity, an areal capacity of 2.22 mAh cm^−2^, and a coating load of 150 g m^−2^ per side. A 25 µm separator foil with a porosity of 42% was wrapped twice around the single anode–cathode pair (one anode, one cathode). All assembly steps were performed in an argon-filled glovebox. After stacking and wrapping, the pouch was vacuum-sealed inside the glovebox. Electrolyte filling was performed with an 80% excess, corresponding to 3 mL (3.6 g) of electrolyte, 1 M LiPF_6_ in 30 : 70 (% w/w) EC/EMC (E-Lyte VP1077)—*cf.*Fig. 6; EMC is ethyl methyl carbonate, C_4_H_8_O_3_ (l). Drying was carried out at 80 °C under vacuum (50–100 mbar) for 12 h in 10 pressure cycles each. Formation was conducted using a CC–CV (constant current, constant voltage) protocol at 0.1 C (17.7 mA) to 3.65 V, followed by a CV step until the current decreased to 0.02 C (3.54 mA). The cells were then discharged at 0.1 C to 2.5 V at 30 °C for three cycles. Based on the limiting cathode, the nominal capacity of each pouch cell was 177 ± 2 mAh.

Calibration data for optimization of the OPM-based shuttling setup and coin-cell electrolyte quantification (Fig. 3) were collected using 1.5 mL cylindrical glass calibration vials with interior dimensions approximately 10 mm (diameter) by 20 mm (height), filled with an off-the-shelf LiPF_6_ solution (Sigma-Aldrich 809357, 2 M LiPF_6_ in EC/DMC 50 : 50 (v/v), battery grade). For additional OPM-detected calibration studies (Fig. 7 and S6D) the same vials were filled with homemade solutions prepared in a nitrogen-filled glovebox from individual components sourced from Sigma-Aldrich (LiPF_6_ salt; EC, DMC, and DEC solvents); these solutions were subsequently transferred to the 5 mm NMR tubes for benchtop measurements. For collection of electrolyte calibration spectra with the SQUID-based setup (Fig. 6), a 20 mL glass vial was filled with the same off-the-shelf solution used to fill the larger-format pouch cells: E-Lyte VP1077, 1 M LiPF6 in 30 : 70 (% w/w) EC/EMC.

### Data processing

The production of frequency spectra from the raw magnetometer (OPM and SQUID) time traces was carried out using MATLAB according to a general procedure outlined in previous work,^26^ as further detailed in the SI. Additional postprocessing of the OPM-detected spectra for lineshape correction, peak phasing, and integration/quantification was implemented in Mathematica (see SI). These spectra were then phased by joining sections with different zeroth-order phases, described in the caption of Fig. 2.

## Conclusions

We have demonstrated the ability of ultralow-field NMR spectroscopy to directly characterize battery electrolyte composition through battery housing, in a manner compatible with nondestructive measurements of functional rechargeable cells. NMR signals were recorded using quantum magnetometers, and a theoretical framework for interpretation of spectra was presented. Time dependence of the electrolyte signals was also tracked to demonstrate relative stability or leakage of sample cells. The quantification and characterization of electrolytes is crucial for the diagnosis of battery defects and aging processes. This technique is shown to be compatible with a range of common electrolytes and does not have fundamental limitations of battery geometry. Nondestructive battery diagnostics remain limited especially under noninvasive open-circuit conditions, and the addition of this method provides critical characterization capability for battery development and testing, as well as second-life assessment.

Given the flexibility and tunability of ZULF-NMR systems, we believe that our results pave the way to functional measurements of commercial pouch or prismatic cells. Through the use of two different ZULF instruments in this work, we have already demonstrated the versatility of the methodology in terms of *e.g.* sample size/shape, experimental geometry, and protocol (shuttling *versus in situ*). The two detection elements, OPMs and SQUIDs, are complementary technologies each with their own advantages—OPMs have the edge for real-world applications requiring portable non-cryogenic sensors,^40^ and SQUIDs for more fundamental studies requiring a larger bandwidth and ability to operate in ambient fields without loss of sensitivity.^52^ To the best of our knowledge, this is the first publication on the topic of ultralow-field NMR to include both OPM- and SQUID-detected experimental data in the same work.

Going forward, envisioned experimental enhancements for diagnostic applications using OPM-based instrumentation with mechanical shuttling include optimization of the measurement duty cycle for faster sensitive detection of electrolyte (Fig. S9), reduction of the sensor offset distance using customized sensors, and optimization of the shuttling field profile to maximize SNR. One may also use a superconducting magnet (up to 20 T) for prepolarization to immediately achieve a 20-fold boost in signal (while still detecting at ultralow field) and speed up data collection by a factor of 400. Signal enhancement is also attainable through measurement of larger volumes of electrolyte, as well as optimization of spectrometer sensitive regions for measurement of specific battery geometries. However, the ability of our method to detect even tens of µL of electrolyte suggests that spatially resolved measurement of larger batteries—through the use of multiple sensors or a scanning system—is also feasible. Protocols without mechanical shuttling might also be explored, through the use of switchable magnets^54,55^ or *in situ* polarization with a solenoid^39^ or other coil system (as in the SQUID-based setup), to enable localized measurements of heavier batteries. For larger batteries unable to fit inside an apparatus, scanning the surface using a prepolarizing-magnet and sensor combination would allow for spatially resolved characterization of the electrolyte, with a sensor array enabling more rapid measurement over the surface area.

In future studies, the low-amplitude *J* peaks (Fig. 6) may also be detected indirectly *via* prior population transfer, whereby one resonantly irradiates *J* transitions and subsequently detects a signal enhancement of the nZF peaks. Such an approach may further enable the identification of chemical species and degradation products in the electrolyte (Fig. S13). Additional diagnostic potential is attainable from measurement of spectral linewidths. Cathode degradation may occur due to transition metal dissolution, and the presence of paramagnetic Ni^2+^ and Mn^2+^ ions in solution could be identified *via* their effect on spectral line broadening.^56^ In general, relaxation properties of spectral lines may be used to better authenticate and discriminate different components of the electrolyte, as well as physical properties such as diffusion and viscosity.^57,58^ This can be achieved by measuring not only linewidths but also the magnetization decay rate for different resonance lines (*T*_1_ relaxation, Fig. S14)—an approach particularly useful in cases of spectral crowding or insufficient resolution. Finally, cycling of battery cells is expected to be associated with additional degradation and consumption of the electrolyte during cycling. For example, cracking of the cathode material can expose fresh surface area with which the electrolyte reacts, thereby consuming the electrolyte^19^ and affecting the spectral signature. All these processes will require careful study to disentangle their various spectral contributions.

Within the framework of nondestructive measurement of electrolyte spectra through battery housing, a wide variety of ULF-NMR or MRI experimental geometries and protocols are realizable for practical applications. The shuttling setup used here was assembled from equipment available in our labs with a total value on order 10k€;^26^ cheaper systems are also possible.^33^ Total cost and complexity of the apparatus depends largely on the choice of detectors and prepolarization technique, but affordable commercial options exist.^40^ Thus, ZULF-NMR shows promise as a complement to other more invasive battery-diagnostic techniques.

## Author contributions

Conceptualization: AMF, RPF, GJR, JWB, JE, KS, DAB, AJ; methodology: AMF, RPF, GJR, RKi, ML, PML, DAB, RKö, AJ; investigation: AMF, RPF, FT, GJR, RKi, WE, RKö; formal analysis, software, visualization: AMF, RPF, FT, RKi, RKö; resources: GJR, ML, PML, RAH, PGB, PK; supervision: DAB, DB, AJ; writing—original draft: AMF, RPF, FT, GJR, ML, AJ; writing—review & editing: AMF, RPF, FT, GJR, RK, WE, PML, RAH, PGB, JWB, JE, KS, RKö, DB, DAB, AJ.

## Conflicts of interest

Authors have filed the patent WO 2025/245002, “Battery electrolyte characterization using ultralow-field NMR”.

## Supplementary Material

SC-017-D5SC04419G-s001

## Data Availability

Data from measured samples, as well as the MATLAB processing code used for production of NMR spectra, is available in an online repository at https://osf.io/rny7b. Supplementary information (SI): supplementary text, Fig. S1–S14, references. See DOI: https://doi.org/10.1039/d5sc04419g.

## References

[cit1] Grey C. P., Dupré N. (2004). Chem. Rev..

[cit2] Trease N. M., Zhou L., Chang H. J., Zhu B. Y., Grey C. P. (2012). Solid State Nucl. Magn. Reson..

[cit3] Pecher O., Carretero-González J., Griffith K. J., Grey C. P. (2017). Chem. Mater..

[cit4] Liu X., Liang Z., Xiang Y., Lin M., Li Q., Liu Z., Zhong G., Fu R., Yang Y. (2021). Adv. Mater..

[cit5] Chandrashekar S., Trease N. M., Chang H. J., Du L.-S., Grey C. P., Jerschow A. (2012). Nat. Mater..

[cit6] Ilott A. J., Trease N. M., Grey C. P., Jerschow A. (2014). Nat. Commun..

[cit7] Ilott A. J., Mohammadi M., Chang H. J., Grey C. P., Jerschow A. (2016). Proc. Natl. Acad. Sci. U. S. A..

[cit8] Ilott A. J., Mohammadi M., Schauerman C. M., Ganter M. J., Jerschow A. (2018). Nat. Commun..

[cit9] Hu Y., Iwata G. Z., Mohammadi M., Silletta E. V., Wickenbrock A., Blanchard J. W., Budker D., Jerschow A. (2020). Proc. Natl. Acad. Sci. U. S. A..

[cit10] Bason M. G., Coussens T., Withers M., Abel C., Kendall G., Krüger P. (2022). J. Power Sources.

[cit11] Pollok S., Khoshkalam M., Ghaffari-Tabrizi F., Kurnia F., Wang D., Li S., Bucher D. B., Rupp J. L. M., Christensen D. V. (2025). Nat. Commun..

[cit12] Romanenko K., Kuchel P. W., Jerschow A. (2020). Chem. Mater..

[cit13] Zhang X., Chatzidrosos G., Hu Y., Zheng H., Wickenbrock A., Jerschow A., Budker D. (2021). Appl. Sci..

[cit14] Zhao K., Wan X., Lin Y., Wu H., Tan X., Zou S., Zhu M., Liu J. (2025). Adv. Energy Mater..

[cit15] Wan X., Xu X., Li F., Song X., Peng C., Liu J. (2024). Small Structures.

[cit16] Wei Y., Wang M., Zhang M., Cai T., Huang Y., Xu M. (2025). Electrochem. Energy Rev..

[cit17] Taskovic T., Adamson A., Clarke A., Alter E. D., Eldesoky A., Gering K. L., Tuul K., Dahn J. R. (2023). J. Electrochem. Soc..

[cit18] Ye Z., Hijazi H., Black W., Azam S., Dahn J. R., Metzger M. (2024). J. Electrochem. Soc..

[cit19] Birkl C. R., Roberts M. R., McTurk E., Bruce P. G., Howey D. A. (2017). J. Power Sources.

[cit20] Leifer N., Aurbach D., Greenbaum S. G. (2024). Prog. Nucl. Magn. Reson. Spectrosc..

[cit21] Kwade A., Haselrieder W., Leithoff R., Modlinger A., Dietrich F., Droeder K. (2018). Nat. Energy.

[cit22] Walder B. J., Conradi M. S., Borchardt J. J., Merrill L. C., Sorte E. G., Deichmann E. J., Anderson T. M., Alam T. M., Harrison K. L. (2021). Sci. Adv..

[cit23] Benders S., Mohammadi M., Klug C. A., Jerschow A. (2020). Sci. Rep..

[cit24] Wu B., Aspers R. L., Kentgens A. P., Zhao E. W. (2023). J. Magn. Reson..

[cit25] Burueva D. B., Eills J., Blanchard J. W., Garcon A., Picazo-Frutos R., Kovtunov K. V., Koptyug I. V., Budker D. (2020). Angew. Chem., Int. Ed..

[cit26] Fabricant A. M., Put P., Barskiy D. A. (2024). Front. Plant Sci..

[cit27] Blanchard J. W., Budker D. (2016). eMagRes.

[cit28] Blanchard J. W., Budker D., Trabesinger A. (2021). J. Magn. Reson..

[cit29] Tayler M. C., Ward-Williams J., Gladden L. F. (2018). J. Magn. Reson..

[cit30] Put P., Pustelny S., Budker D., Druga E., Sjolander T. F., Pines A., Barskiy D. A. (2021). Anal. Chem..

[cit31] Alcicek S., Put P., Kubrak A., Alcicek F. C., Barskiy D., Gloeggler S., Dybas J., Pustelny S. (2023). Commun. Chem..

[cit32] Hartwig S., Albrecht H. H., Scheer H. J., Burghoff M., Trahms L. (2013). Appl. Magn. Reson..

[cit33] Tayler M. C., Theis T., Sjolander T. F., Blanchard J. W., Kentner A., Pustelny S., Pines A., Budker D. (2017). Rev. Sci. Instrum..

[cit34] Kovtunov K. V., Pokochueva E. V., Salnikov O. G., Cousin S. F., Kurzbach D., Vuichoud B., Jannin S., Chekmenev E. Y., Goodson B. M., Barskiy D. A., Koptyug I. V. (2018). Chem.–Asian J..

[cit35] Eills J., Budker D., Cavagnero S., Chekmenev E. Y., Elliott S. J., Jannin S., Lesage A., Matysik J., Meersmann T., Prisner T., Reimer J. A., Yang H., Koptyug I. V. (2023). Chem. Rev..

[cit36] Blanchard J. W., Sjolander T. F., King J. P., Ledbetter M. P., Levine E. H., Bajaj V. S., Budker D., Pines A. (2015). Phys. Rev. B.

[cit37] Jiang M., Wu T., Blanchard J. W., Feng G., Peng X., Budker D. (2018). Sci. Adv..

[cit38] Eills J., Picazo-Frutos R., Bondar O., Cavallari E., Carrera C., Barker S. J., Utz M., Herrero-Gómez A., Marco-Rius I., Tayler M. C. D., Aime S., Reineri F., Budker D., Blanchard J. W. (2023). Anal. Chem..

[cit39] Bodenstedt S., Mitchell M. W., Tayler M. C. D. (2021). Nat. Commun..

[cit40] Osborne J. , Orton J., Alem O. and Shah V., Proc. SPIE 10548, Steep Dispersion Engineering and Opto-Atomic Precision Metrology XI, 2018, 10548G, 51

[cit41] Hogben H., Krzystyniak M., Charnock G., Hore P., Kuprov I. (2011). J. Magn. Reson..

[cit42] Appelt S., Häsing F. W., Sieling U., Gordji-Nejad A., Glöggler S., Blümich B. (2010). Phys. Rev. A.

[cit43] Stern Q., Sheberstov K. (2023). Magn. Reson..

[cit44] Hildebrand S., Ferrario F., Lebedeva N. (2024). Energy Technol..

[cit45] Sheng S., Li H., Zhang Y., Li L., Xiao K., Huang X., Liu Y., Xu W., Li Z., Yan L., Yan Z., Huang Y., Sun Q. (2025). Light adv. manuf..

[cit46] Wang Q., Mao B., Stoliarov S. I., Sun J. (2019). Prog. Energy Combust. Sci..

[cit47] Li Z., Cong J., Ding Y., Yang Y., Huang K., Ge X., Chen K., Zeng T., Huang Z., Fang C., Huang Y. (2024). Electrochem. Energy Rev..

[cit48] Fabricant A., Novikova I., Bison G. (2023). New J. Phys..

[cit49] Barskiy D. A., Blanchard J. W., Budker D., Eills J., Pustelny S., Sheberstov K. F., Tayler M. C., Trabesinger A. H. (2025). Prog. Nucl. Magn. Reson. Spectrosc..

[cit50] Kitching J., Shaffer J. P., Budker D. (2025). Optica.

[cit51] Teleanu F. , Fabricant A. M., Zhang C., Centers G. P., Budker D., Barskiy D. A. and Jerschow A., arXiv, 2025, preprint arXiv2511.08517, 10.48550/arXiv.2511.08517

[cit52] Storm J.-H., Hömmen P., Drung D., Körber R. (2017). Appl. Phys. Lett..

[cit53] Voigt J., Knappe-Grüneberg S., Gutkelch D., Haueisen J., Neuber S., Schnabel A., Burghoff M. (2015). Rev. Sci. Instrum..

[cit54] Blümler P., Soltner H. (2023). Appl. Magn. Reson..

[cit55] Vogel M. W., Giorni A., Vegh V., Pellicer-Guridi R., Reutens D. C. (2016). PLoS One.

[cit56] Allen J. P., Grey C. P. (2023). J. Phys. Chem. C.

[cit57] Kim J. S., Wu Z., Morrow A. R., Yethiraj A., Yethiraj A. (2012). J. Phys. Chem. B.

[cit58] Korb J.-P., Vorapalawut N., Nicot B., Bryant R. G. (2015). J. Phys. Chem. C.

