# Supplementary material for: Enabling nondestructive observation of electrolyte composition in batteries with ultralow-field nuclear magnetic resonance
Source: Chem Sci. 2026 Jan 29;17(12):5877–87. doi: 10.1039/d5sc04419g (PMC12915680; doi:10.1039/d5sc04419g)
Supplement: SC-017-D5SC04419G-s001 [file SC-017-D5SC04419G-s001.pdf]

Supplementary Information (SI) for Chemical Science.
This journal is © The Royal Society of Chemistry 2026

Supplementary Information for

Enabling nondestructive observation of electrolyte composition in batteries with ultralow-field nuclear magnetic resonance

A. M. Fabricant, R. Picazo-Frutos, F. Teleanu, G. J. Rees, R. Kircher, M. Lin, W. Evans, P.-M. Luc, R. A. House, P. G. Bruce, P. Krüger, J. W. Blanchard, J. Eills, K. F. Sheberstov, R. Körber, D. Budker, D. A. Barskiy & A. Jerschow

D. Budker & A. Jerschow.
E-mail: budker@uni-mainz.de, alexej.jerschow@nyu.edu

This PDF file includes:

Supplementary Text
Figs. S1 to S14
References

Supplementary Text

Supplementary figures. Figures S1–S14 show additional experimental results and simulations relevant to the material discussed in the main text.

Theory background. The purpose of this section is to provide a basic understanding of the spectral pattern observed for the PF_6^- spin system (Fig. 2). For more complete reviews on zero-to-ultralow-field nuclear-magnetic-resonance (ZULF-NMR) experiments and simulations, the reader is encouraged to consult other recent publications (1, 2). Our investigations were primarily conducted in the ultralow-field (ULF) regime at a background magnetic field $B_z = 2.7\,\mu\mathrm{T}$, such that both Zeeman and J-coupling interactions contribute to nuclear-spin dynamics. The general Hamiltonian describing the coherent evolution of the system is given by

$$\hat{\mathcal{H}}^{\mathrm{ULF}}_{\mathrm{PF}_6} = 2\pi\, J_{\mathrm{PF}}\, \hat{\mathbf{I}} \cdot \sum_i^N \hat{\mathbf{S}}_{\mathbf{i}} + \left(-\gamma_I\, B_z\, \hat{I}_z - \gamma_S\, B_z \sum_i^N \hat{S}_{i,z} \right) \equiv \hat{\mathcal{H}}_J + \hat{\mathcal{H}}_{\mathrm{Zeeman}}\,, \tag{1}$$

where I and S_i represent the ^{31}P and ^{19}F nuclear spins, respectively (here, $N = 6$); $\hat{I}_z$ and $\hat{S}_{i,z}$ are the spin-projection operators along the quantization axis $\hat{z}$ (corresponding to the z-axis in the experimental coordinate system of Fig. 1). The J-coupling constant, $J_{\mathrm{PF}} \approx 710\,\mathrm{Hz}$, is significantly larger than the Larmor frequencies $\nu_{\mathrm{P}} = \gamma_{\mathrm{P}} B_z/2\pi \approx 46\,\mathrm{Hz}$ and $\nu_{\mathrm{F}} = \gamma_{\mathrm{F}} B_z/2\pi \approx 108\,\mathrm{Hz}$, where γ denotes gyromagnetic ratios in (SI units) $\mathrm{rad}\cdot\mathrm{s}^{-1}\cdot\mathrm{T}^{-1}$—such that $\gamma_{\mathrm{P}}/2\pi = 17.2$ Hz/μT and $\gamma_{\mathrm{F}}/2\pi = 40.1$ Hz/μT. Thus, the Zeeman interaction acts as a perturbation $\hat{\mathcal{H}}_{\mathrm{Zeeman}}$ to the eigenstates of the $\hat{\mathcal{H}}_J$ Hamiltonian, lifting the degeneracy inside each eigenstate manifold (Fig. 2 and Fig. S3). Note that Eq. (1) is written such that the Hamiltonian has units of $\hbar\omega$ and nuclear spins have units of $\hbar$, where $\hbar$ is the reduced Planck constant and ω is angular frequency. In this section, we set $\hbar = 1$ for convenience.

In the absence of the external field ($B_z = 0$), there are 128 eigenstates distributed among seven manifolds with the eigenvalues $2\pi\{-2J_{\mathrm{PF}}, -\frac{3}{2}J_{\mathrm{PF}}, -J_{\mathrm{PF}}, 0, \frac{1}{2}J_{\mathrm{PF}}, J_{\mathrm{PF}}, \frac{3}{2}J_{\mathrm{PF}}\}$. These manifolds are characterized by their total spin angular momentum $F = I + S$, where $I = \frac{1}{2}$ (phosphorus) and S is the sum of fluorine spins, while states inside a given manifold are distinguished by the $\hat{z}$-projection of total spin angular momentum, m_F, with values $\{-F, -F+1, \ldots, F\}$. Under ZULF-NMR conditions, transitions between manifolds are observed as J peaks following the selection rules $\Delta F = \pm 1$ and $\Delta S = 0$, as illustrated in Fig. S3—corresponding to peaks at the frequencies $\{\frac{3}{2}J_{\mathrm{PF}}, \frac{5}{2}J_{\mathrm{PF}}, \frac{7}{2}J_{\mathrm{PF}}\}$.

Upon application of the B_z field, the degeneracy in each manifold is lifted and transitions between eigenstates within the same manifold are also observed as the near-zero-frequency (nZF) peaks. These transitions are characterized by the selection rules $\Delta F = 0$, $\Delta S = 0$, and $\Delta m_F = \pm 1$. In addition, the J peaks are shifted to higher frequencies with increasing B_z, corresponding to further splitting of the energy levels. To compute the transition frequencies as a function of field, we use the following expression for the energy of each state (3):

$$\frac{E\,(F,\,S,\,m_F)}{2\pi} = m_F\,\nu_F - \frac{J_{\mathrm{PF}}}{4} + S\sqrt{\left(F + \frac{1}{2}\right)^2 J_{\mathrm{PF}}^2 + 2\,m_F\,J\,(\nu_{\mathrm{P}} - \nu_{\mathrm{F}}) + (\nu_{\mathrm{P}} - \nu_{\mathrm{F}})^2}\,, \tag{2}$$

By performing a Taylor expansion in B_z (around $B_z = 0$) and keeping only first-order terms, we arrive at approximate expressions for both the δ_m^{nZF} and δ_m^{J} frequencies, which are in agreement with spectra obtained by numerical simulation in Spinach (4) (Fig. S3):

$$\delta_m^{\mathrm{nZF}} \approx \frac{\gamma_{\mathrm{P}} + m \times \gamma_{\mathrm{F}}}{2\pi\,(m+1)} B_z\,, \quad \delta_m^{J} \approx \frac{m}{2} J_{\mathrm{PF}} + \frac{\gamma_{\mathrm{F}} - \gamma_{\mathrm{P}}}{2\pi m} B_z\,. \tag{3}$$

When further increasing B_z, i.e. the solenoid field, the splitting pattern gains complexity as quadratic terms become significant (Fig. S4). The choice of detection field is thus motivated by a balance between maximizing SNR and working in a noise-free region where peak integration can be performed for quantitative analysis. Notably, the J peaks have much lower intensities than the nZF peaks (Fig. 6), motivating our choice to focus on the latter for detection and quantification of the $LiPF_6$ electrolyte properties.

Relationship between absolute signal and concentration. To investigate the absolute relationship between molecular concentrations and NMR signals measured with the OPM-based spectrometer, theoretical calculation of expected magnetic fields was carried out as follows. For calibration, we first considered the simplest case of a pure water sample prepolarized at $B_p = 1\,\mathrm{T}$ and room temperature (20°C). Under these typical experimental conditions, the expected thermal prepolarization of proton spins is (5, 6)

$$P_{\mathrm{therm}} = \frac{\gamma_{^1\mathrm{H}}\,\hbar\,(\mathcal{I}+1)\,B_{\mathrm{P}}}{3\,k_{\mathrm{B}}T} = \frac{(\gamma_{^1\mathrm{H}}/2\pi)\,h\,B_{\mathrm{P}}}{2\,k_{\mathrm{B}}T}\,, \tag{4}$$

where the proton has gyromagnetic ratio $\gamma_{^1\mathrm{H}}/2\pi = 42.6$ Hz/μT and nuclear-spin quantum number $\mathcal{I} = 1/2$, h is the Planck constant, and k_B is the Boltzmann constant. This expression yields

$$P_{\mathrm{therm}} = \frac{\left(42.6 \times 10^6\,\mathrm{Hz}\cdot\mathrm{T}^{-1}\right)\left(6.63 \times 10^{-34}\,\mathrm{J}\cdot\mathrm{s}\right)(1\,\mathrm{T})}{2\left(1.38 \times 10^{-23}\,\mathrm{J}\cdot\mathrm{K}^{-1}\right)(293\,\mathrm{K})} = 3.5 \times 10^{-6}\,. \tag{5}$$

Given the molar concentration of water protons, $C = 2 \cdot 55\,\mathrm{M} = 110\,\mathrm{M}$, the amplitude of sample magnetization (magnetic moment per unit volume) can then be estimated as

$$\begin{aligned} M &= C \cdot N_A \cdot \gamma_{^1\mathrm{H}}\,\hbar\, I \cdot P_{\mathrm{therm}} \\ &= \left(110\,\mathrm{mol}\cdot\mathrm{L}^{-1}\right)\left(6.02 \times 10^{23}\,\mathrm{mol}^{-1}\right)\left(42.6 \times 10^6\,\mathrm{Hz}\cdot\mathrm{T}^{-1}\right)\left(6.63 \times 10^{-34}\,\mathrm{J}\cdot\mathrm{s}\right)\left(\frac{1}{2}\right)\left(3.5 \times 10^{-6}\right) \\ &= 3.3 \times 10^{-6}\ \mathrm{A}\cdot\mathrm{m}^2\cdot\mathrm{L}^{-1} \\ &= 3.3 \times 10^{-3}\ \mathrm{A}\cdot\mathrm{m}^{-1}\,, \end{aligned} \tag{6}$$

where N_A is the Avogadro constant. For a sample of volume $V = 80\,\mu\mathrm{L}$ (the estimated interior volume of the battery cells), the magnetic moment is then $m = M \cdot V = 2.6 \times 10^{-10}\ \mathrm{A}\cdot\mathrm{m}^2$. The ideal sample geometry would be that of a uniformly magnetized sphere, giving rise to a pure dipole magnetic field outside. Ignoring any relaxation mechanisms between polarization and measurement which might affect M, the maximum gradiometric quadrature amplitude (7) measured at the sensor offset distance 17.5 mm from the center of the sample is therefore

$$B = \frac{3\,\mu_0\, m}{2\pi r^3} = \frac{3\left(4\pi \times 10^{-7}\,\mathrm{H}\cdot\mathrm{m}^{-1}\right)\left(2.6 \times 10^{-10}\,\mathrm{A}\cdot\mathrm{m}^2\right)}{2\pi\,(0.0175\,\mathrm{m})^3} \approx 30\,\mathrm{pT}\,. \tag{7}$$

Here we have used the vacuum magnetic permeability μ_0 and reported the magnetic-field amplitude to one significant figure. For $V = 1.5\,\mathrm{mL}$, the volume of our calibration vials, the expected field from a uniformly magnetized spherical sample is approximately 500 pT. This amplitude is robust with respect to temperature fluctuations of the sample, varying only a few percent per 10°C.

Such calculations are easily extendable to other chemical systems. For the case of the solvent 50:50 (v/v) EC/DMC, containing five protons per molecule, the molar concentration of protons is $5 \times 13.4 = 67.0\,\mathrm{M}$. Thus, Eq. (7) is scaled by the factor 67/110.

In actual experiment using the shuttling setup, samples have a cylindrical geometry (see Experimental section of the main text) and the initial magnetization M_0 decays on a timescale characterized by the longitudinal spin-relaxation time, T_1, according to $M = M_0 \cdot \exp(-t/T_1)$, where t denotes the time since leaving the prepolarization field. Taking these effects into account for measurement of a transversely polarized cylindrical sample ((7, 8) and Fig. S14), we find for the calibration vial an expected water-proton signal of 400 pT. In our experimental geometry where the sensor offset distance is large relative to the sample radius, signal decay due to relaxation (during shuttling and pulse application, Fig. 1) tends to dominate any losses resulting from nonspherical sample geometry. Even though we have not considered possible effects of nonuniform magnetization or mechanical motion of samples, we do measure water-proton calibration signals of the expected order of magnitude, and the EC/DMC proton signal is indeed smaller by about a factor of two (Fig. S6). By contrast, the measured solvent-proton signals from the sample cells (Fig. 3B) are consistently at least an order of magnitude less than the theoretical value of 20 pT. This discrepancy could be explained by systematic effects, including spurious magnetic fields due to eddy currents induced by shuttling conductive material through external field gradients (Fig. S5), which may negatively impact sample magnetization. As an additional check, we filled an empty battery casing with 74 μT of deionized water (verified by weighing before and after filling) and obtained a proton signal of 3 pT (Fig. S7B). According to the vial calibration data in Fig. S6, this would correspond to an expected solvent-proton signal of 1 pT in the electrolyte cells, which is close to the largest measured value of 800 fT (Fig. 3).

The above considerations underscore the advantages of our method for calculation of electrolyte concentrations based on ratios of spectral peaks (see main text), which is immune to any systematics affecting all peaks equally. This attribute is critical especially in the ZULF-NMR experimental modality, where, in contrast to conventional higher-field NMR measurements, the sample does not fill the entire sensitive region of the spectrometer. Furthermore, electrolyte leakage due to poor sealing during sample production can be investigated and diagnosed through study of time-resolved peak dynamics (Fig. S8), which also allows for observation of differing compound volatilities.

The approach presented here is also suitable for applications where the sample volume and/or geometry is unknown, as may occur in real-world battery diagnostics.

Data sets and analysis. Processing of raw OPM time-series data in MATLAB proceeded according to the protocol published in (7). To enhance SNR of spectra from the sample cells, the first 5 s of the acquired time series were used, $\sim 10\,000$ scans were averaged in the time domain, and a 300 Hz low-pass filter was applied in the frequency domain. A zeroth-order global phase of $e^{i\phi}$ ($\phi = \frac{9}{10}\pi$ for the typical case where the first 25 ms of the time series were dropped to eliminate pulse-related artifacts) was applied to the complex fast Fourier transform (FFT) to obtain an absorptive proton peak in the real part of the spectrum (Figs. S5–S7). Subsequent postprocessing in Mathematica encompassed the following steps:

1. Baseline cleaning of the four differently phased versions of the spectrum (see main text) in the region 51–391 Hz. This was achieved by fitting the real part of a mathematical model to the real part of each phased spectrum. The fit function was the sum of six complex Lorentzians (including the solvent-proton peak, the four largest PF_6^- peaks, and a lower-frequency noise background) as well as a polynomial up to second order. Spectral regions containing major sharp noise peaks including power-line noise and associated harmonics were excluded from the fitting. The corresponding fit, excluding the peaks of interest, was then subtracted from each phased spectrum.

2. Joining of the four phased spectra at 91.4, 104.9, and 123.8 Hz, to obtain a fully absorptive concatenated spectrum for display purposes (Fig. 2).

3. Integration of two 8 Hz spectral regions centered on the two largest PF_6^- peaks and the proton-solvent peak, to quantify the solute and solvent signals (Fig. 3). In order to reduce errors associated with baseline variation, a line fitted to the endpoints of the integration window was first subtracted from the spectrum.

4. Calculation of electrolyte concentrations according to the procedure detailed in the main text. Although error bars on measured signals from sample 7 were obtained by taking the standard error of repeated measurements (Fig. 4), it was necessary to scale these for other samples according to SNR, such that larger SNR would correspond to smaller error bars. For this purpose, we assumed a simple linear dependence of standard error on signal (since the noise level was generally stable across all experiments). Specifically, after calculating the standard error on the mean (SEM) for both the solute and solvent peaks of the sample-7 spectrum, we used a linear fit to these two data points to relate the signal $\mathcal{S}$ (area in pT) of any measured peak to an associated SEM: $\mathcal{S} = 0.00281 + 0.00653 \times \text{SEM}$, where the SEM corresponds to the error bar on a measured signal $\mathcal{S}$.

Time-series data from the SQUID gradiometer were collected at 30 kΩ feedback resistance corresponding to a magnetic-field conversion of 138 pT/V and processed using MATLAB. In the time domain, a scalar background was removed from the data and a Hanning window was implemented. Using zero-filling of 2^{16} to increase spectral resolution, an FFT was applied to generate the magnitude frequency spectrum. Given the short (5 s) acquisition time, it was desirable to apply lineshape correction to electrolyte solution spectra (Fig. 6)—data were apodized in the time domain (multiplication by a decaying exponential $e^{-t/0.3}$). For proton spectra of pouch-cell batteries (Fig. S11D), no apodization was applied but a 300 Hz low-pass filter was used for SNR improvement, and a 150 mHz moving average was used for plotting.

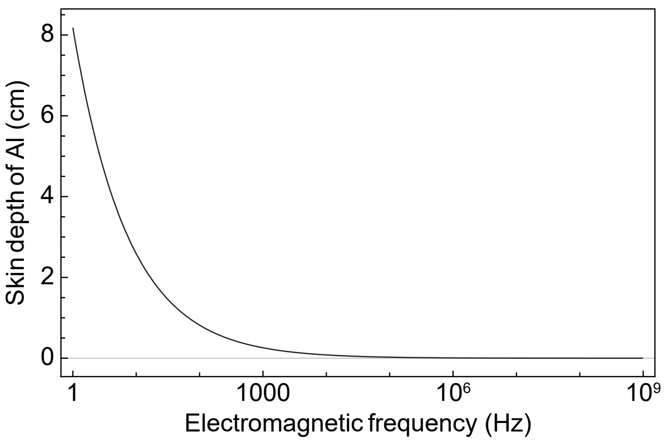

Fig. S1. Simulated skin depth of electromagnetic radiation in aluminum as a function of frequency $\omega/2\pi$ (log-linear plot), according to a standard empirical formula which scales approximately as $1/\sqrt{\omega}$. Typical literature values of Al material parameters were used: resistivity $\rho = 2.65 \times 10^{-8}\,\Omega \cdot$ m, permeability $\mu = 1.26 \times 10^{-6}$ H $\cdot$ m^{-1}, and permittivity $\varepsilon = 4 \times 10^{-11}$ C$^2 \cdot$ N$^{-1} \cdot$ m^{-2}.

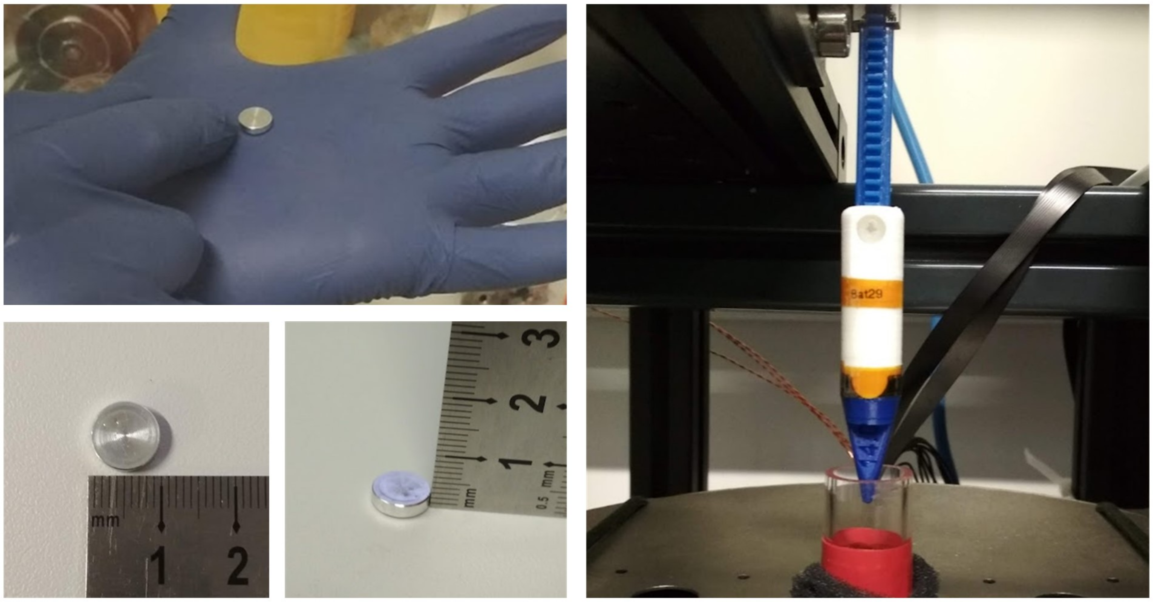

Fig. S2. Photographs of the coin-cell samples and sample holder prior to measurement in the OPM-based shuttling setup (cf. Fig. 1).

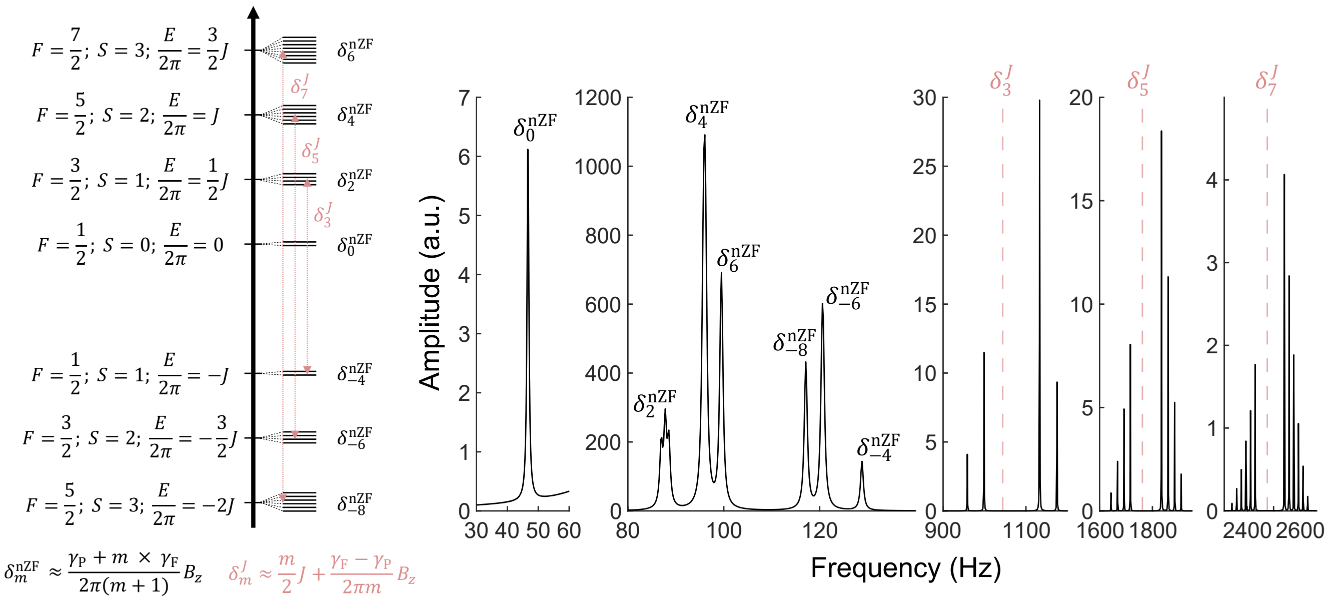

Fig. S3. Left: Energy-level diagram for the PF_6^- system in the ULF regime (energies are expressed in Hz, considering $h = 1$). The Zeeman interaction lifts the degeneracy inside manifolds of total spin angular momentum F, leading to transitions between states inside the same manifold observed as near-zero-frequency (nZF) peaks δ_m^{nZF}. The selection rules for these transitions are $\Delta F = 0$, $\Delta S = 0$, $\Delta m = \pm 1$, where S is the sum of fluorine nuclear spins. The J peaks δ_m^J correspond to transitions between different manifolds that follow the selection rules $\Delta F = \pm 1$, $\Delta S = 0$ and are centered around frequencies calculated using the derived equations (see SI text); for $B_z = 2.7\,\mu$T, we have $\delta_3^J = 1087$ Hz, $\delta_5^J = 1790$ Hz, and $\delta_7^J = 2497$ Hz using $J = J_{\mathrm{PF}} = 710$ Hz. Right: Simulated ULF-NMR spectra using the Spinach program in MATLAB based on experimental parameters of the shuttling setup and protocol (Fig. 1), with peaks corresponding to the labeled transitions. In actual experiment, the phosphorus peak close to 50 Hz is dominated by the much stronger lithium precession peak (Fig. S6). We note that the precise value of J_{PF} depends on solvent composition (9, 10). For $LiPF_6$ in 30:70 (% w/w) EC/EMC, we found $J_{\mathrm{PF}} = 707.4$ Hz based on a SQUID measurement at zero field (c.f Fig. 6); for $LiPF_6$ in 50:50 (v/v) EC/DMC, we found $J_{\mathrm{PF}} = 707.7$ Hz using a benchtop spectrometer (cf. Fig. S13B).

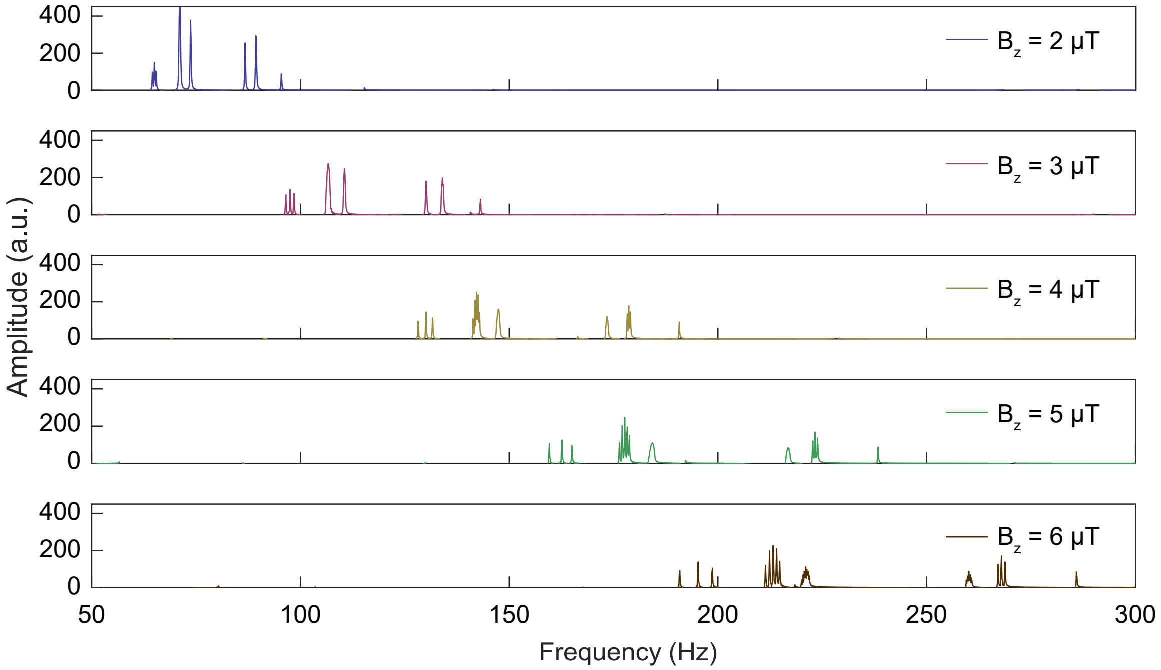

Fig. S4. Simulated ULF-NMR magnitude spectrum of the PF_6^- spin system as a function of background magnetic field B_z following the approach described in the SI text and Fig. S3, motivating the use of lower fields to avoid further splitting of the spectral lines which in turn would reduce the overall SNR of individual peaks (Fig. 2).

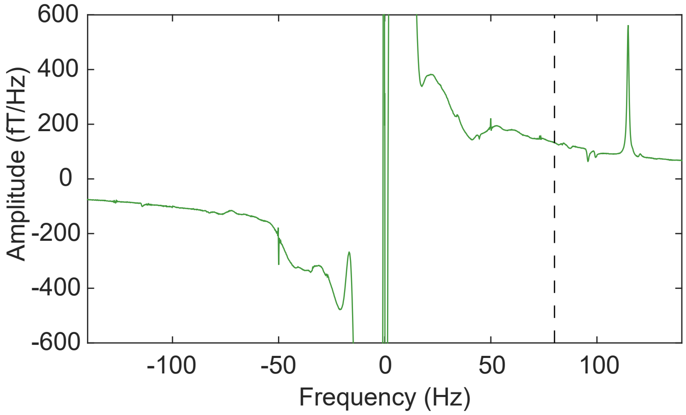

Fig. S5. Experimental spectrum (real part of the complex fast Fourier transform, FFT) showing characteristic low-frequency noise of a sample coin cell (sample 7), attributed to eddy currents induced by mechanical shuttling of the metallic battery housing from high to low magnetic field (Fig. 1). See Fig. 2 for the postprocessed (phased and baseline-corrected) spectrum in the region 80–140 Hz, here delineated with a dashed vertical line. The observed frequency dependence of the noise floor motivates the use of a detection field in the microtesla range (2.7 μT), shifting the solvent proton peak above 100 Hz. Due to the positive gyromagnetic ratios of both the proton and PF_6^- spin systems, signals only appear in the positive part of the spectrum using the quadrature detection method (7).

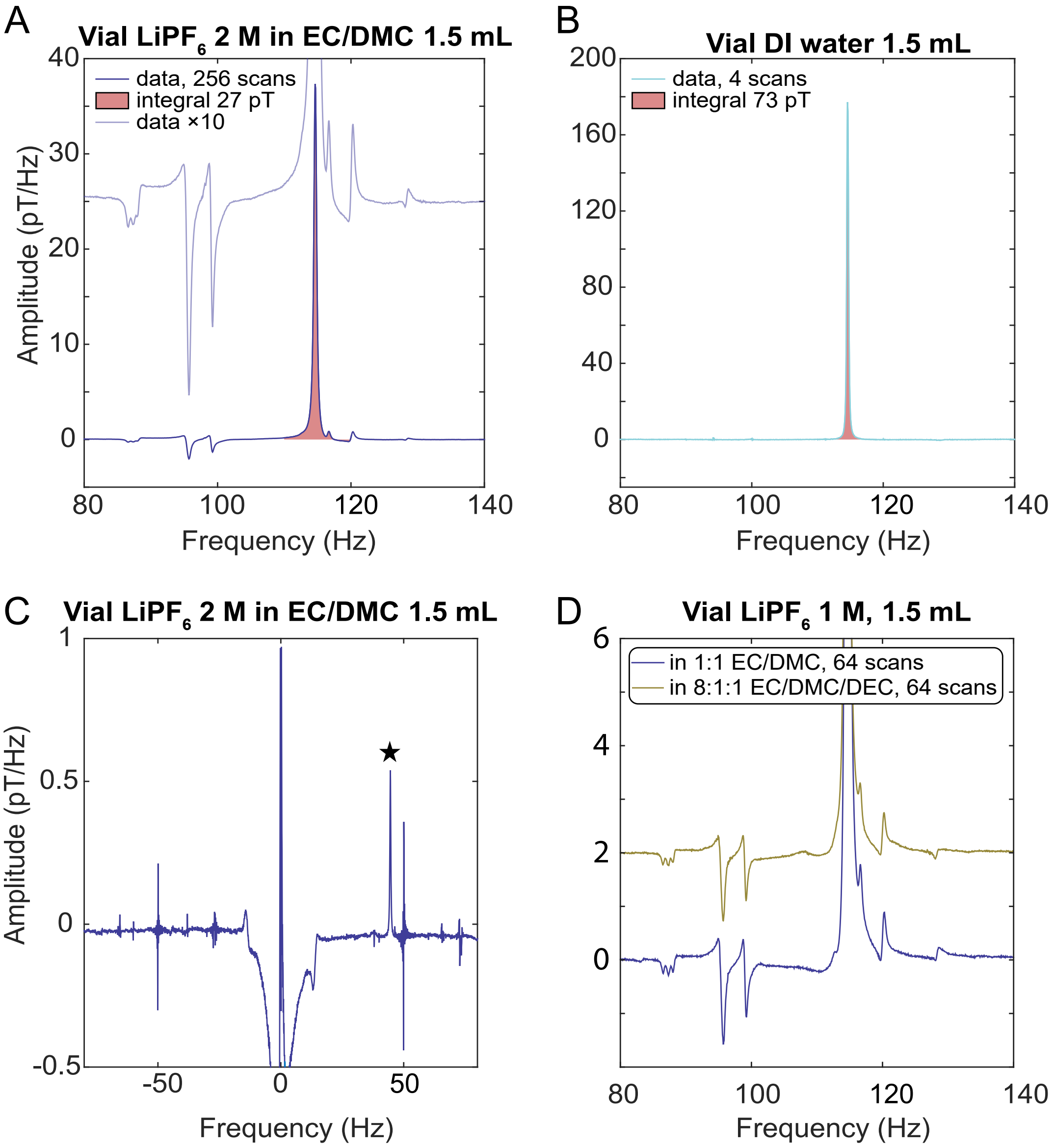

Fig. S6. Comparison of calibration data collected using the OPM-based shuttling setup (Fig. 1), for equal volumes of (A) a preparation of 2 M $LiPF_6$ in 1.5 mL of 50:50 (v/v) EC/DMC (Sigma-Aldrich) and (B) deionized (DI) water; the water-proton signal is approximately three times larger than the solvent-proton signal—compare Fig. S6. Both plots show the real part of the complex FFT; the different phases of peaks in these raw spectra arise from removal of the first 25 ms of the free-induction-decay (FID) signal, which contains artifacts due to the sensors recovering from application of the magnetic-field pulse (Fig. 1C). Panel (C) corresponds to the same data set as (A), zooming in on the ^{7}Li resonance at 45 Hz (starred, next to 50 Hz power-line noise peak). Because ^{7}Li, like ^{1}H and PF_6^-, has a positive gyromagnetic ratio ($\gamma_{^7\mathrm{Li}}/2\pi \approx 17\,\mathrm{Hz}/\mu\mathrm{T}$), the lithium peak only appears in the positive part of the quadrature spectrum. The near-DC features are mainly due to mechanical noise. (D) Comparative test demonstrating that the PF_6^- ULF signature in a 1 M $LiPF_6$ sample remains stable under change of electrolyte composition from 50:50 (v/v) EC/DMC to 80:10:10 (v/v/v) EC/DMC/DEC. All calibration samples were contained in identical 1.5 mL screw-neck vials (BGB ND8, 32 mm height, 11.6 mm outer diameter).

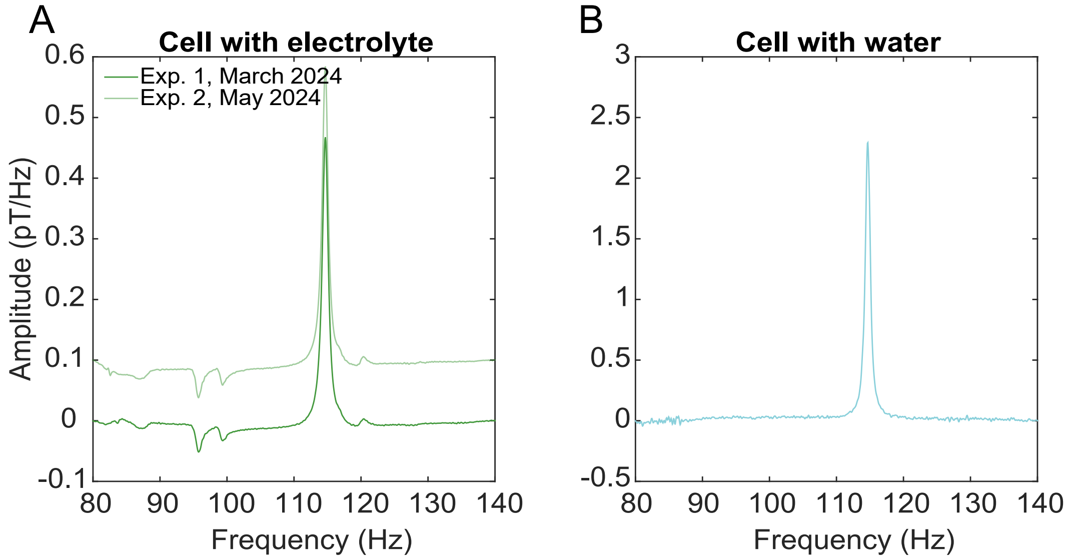

Fig. S7. Comparison of measured raw spectra (real part of the complex FFT) from sample coin cells containing electrolyte or water, cf. Figs. 2–3. Data from OPM sensors were processed according to the protocol described in the SI text and a linear trend was removed to produce the displayed plots. (A) Spectra from an electrolyte battery (sample 7, nominally 2.5 M $LiPF_6$ in 50:50 EC/DMC) recorded in two separate experiments of $\sim$10 000 scans taken two months apart, demonstrating the reproducibility of the measurement and stability of the electrolyte signal in this time frame. The first (darker) spectrum corresponds to the same data set as in Fig. 2; the second (lighter) spectrum is offset vertically for clarity. Integral of the solvent proton peak at 115 Hz is approximately 700 fT, cf. Fig. 3. (B) Spectrum obtained from 64 scans of a sample cell filled with 74 μL of DI water. Integral of the water-proton peak at 115 Hz is approximately 3 pT; the feature at 84 Hz is a noise peak.

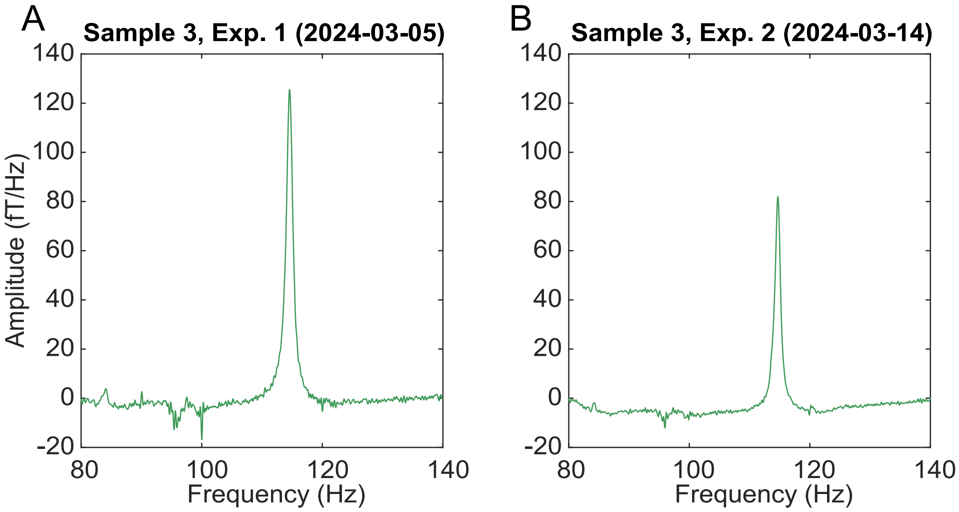

Fig. S8. Comparison of measured raw spectra from the same sample coin cell (sample 3) recorded nine days apart, showing reduction of electrolyte signal associated with leakage; compare Fig. S7A. Spectrum (A) was obtained from 2913 scans while spectrum (B) was obtained from 10 000 scans, which explains the difference in SNR.

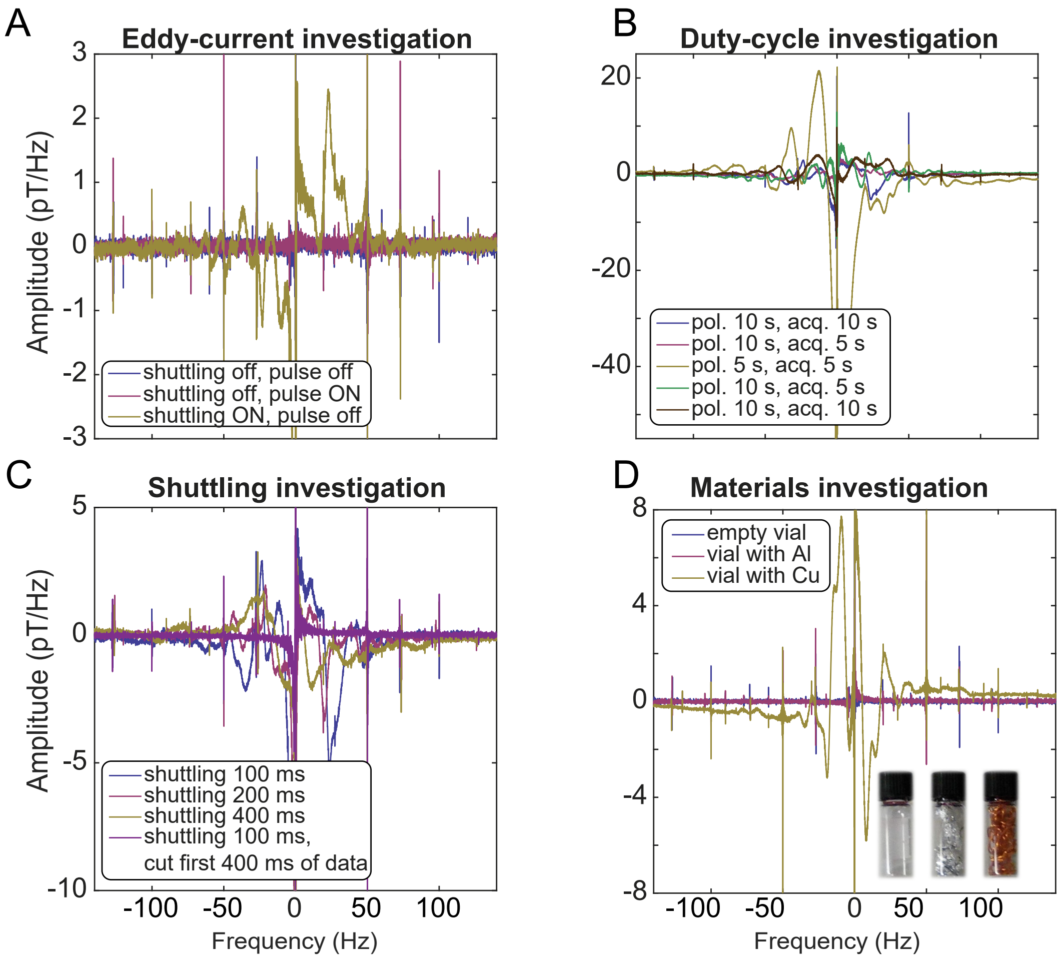

Fig. S9. (A)–(C) Noise spectra (real part of the complex FFT, average of four scans) collected using the shuttling setup from a realistic battery structure without electrolyte but containing all other components, including anode, cathode, separator and current collector—see Results and Discussion in the main text. Although slightly higher baseline noise was observed when shuttling the Cu current collector (from the high- to low-field regions, Fig. 1) in addition to the aluminum housing and other components, this technical challenge can be overcome by dropping more initial points of the measured time series. (A) confirms that baseline noise is primarily attributable to shuttling and not to pulse application. (B) suggests that shortening the acquisition time does not increase baseline noise, while shortening the polarization time does; spectra were collected in the order plotted to account for possible systematics related to buildup of permanent magnetization. (C) shows that reducing shuttling speed does not significantly decrease baseline noise, but dropping the first 400 ms of the time series is effective—this is acceptable in light of the measured spin-relaxation times (Fig. S14). (D) Noise spectra (real part of the complex FFT, average of four scans) from an empty glass vial as well as identical vials filled with either aluminum foil or copper wire, indicating that Cu is a more problematic conductive material than Al in terms of magnetic noise attributed to shuttling-induced eddy currents. Note that because the kitchen-grade foil is not composed of pure Al, it is expected to be less conductive than the AG7 battery cases used in this work (Fig. S5) and therefore to generate less magnetic noise. All spectra were acquired at 2.7 μT with 10 s polarization, 100 ms shuttling, 200 μs pulse, and 10 s acquisition, unless stated otherwise.

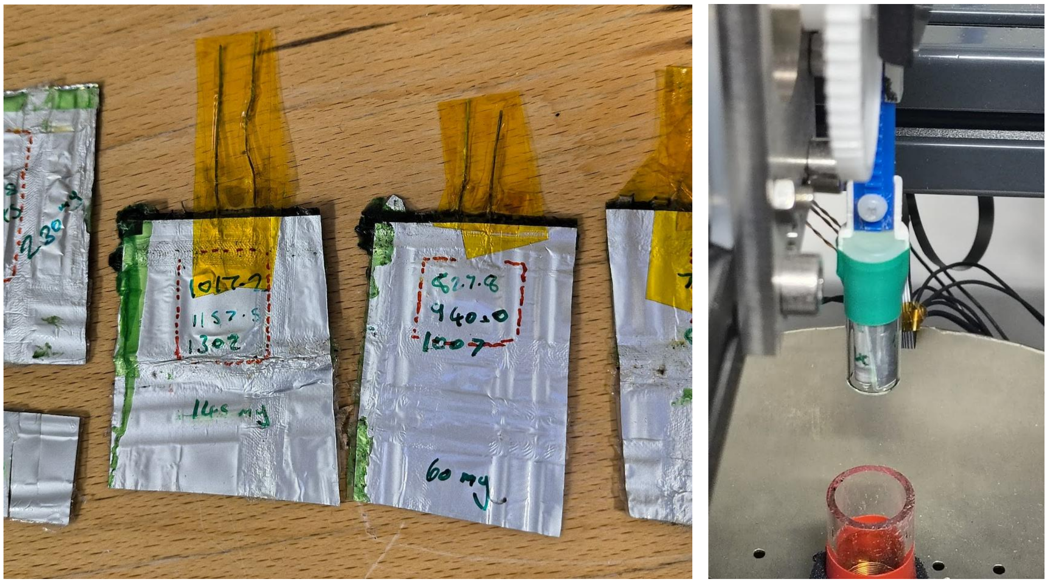

Fig. S10. Examples of functional miniaturized pouch cells manufactured from commercial components for the measurements discussed in the main text and Fig. 5, all of which were found to compatible with ZULF-NMR instrumentation. The active electrolyte region, indicated by red dashed lines, has a width of 9.5 mm. Labeled numbers correspond to the weight of the cell before and after filling with LP30 electrolyte; see main text for details. Right panel shows a pouch cell rolled up inside a sample vial prior to measurement in the shuttling setup. Cells with and without Cu contacts were tested—while mechanical shuttling of the contacts caused some additional spectral baseline noise, measuring farther away from the contacts can bypass this issue.

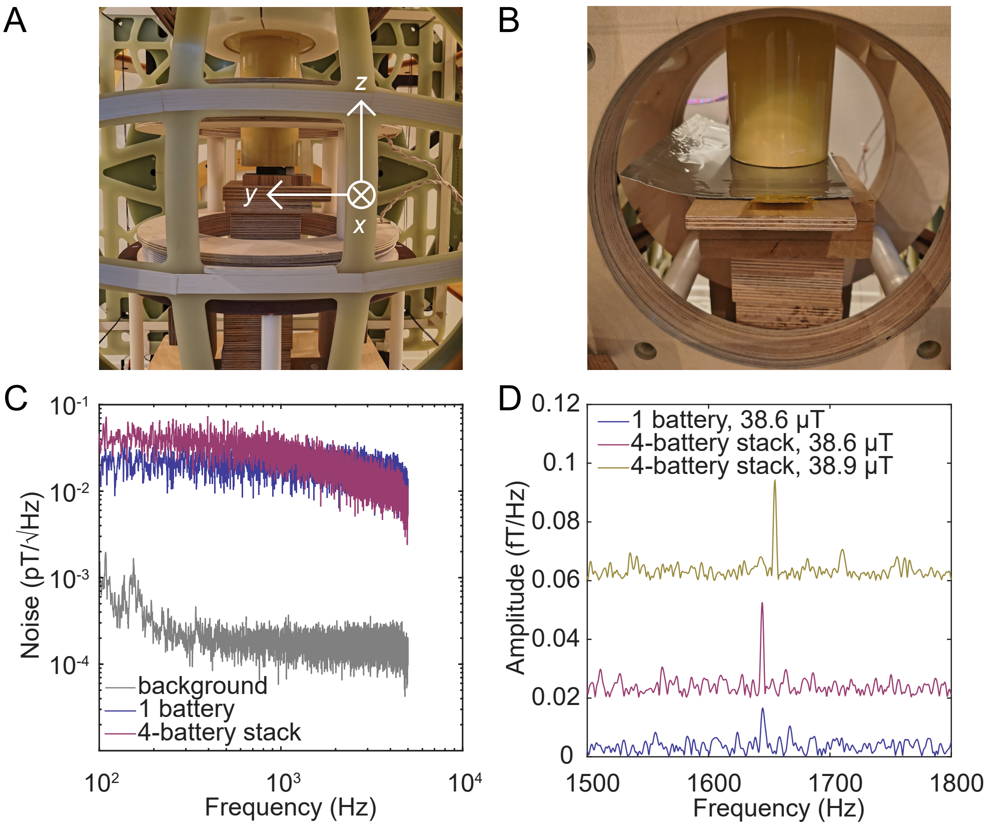

Fig. S11. (A) ZULF-NMR apparatus with SQUID gradiometric detection along the z-direction (11), used for benchmarking measurements as described in the Experimental section of the main text. Photo shows a calibration sample (brown bottle, containing 20 mL of 1 M $LiPF_6$ in EC/EMC) placed under the dewar (yellow circle) at the center of a coil-frame structure. Outer coils produce a tunable detection field along the x-direction, while inner coils can be oriented along either the y- or z- (pictured here) directions to produce a switchable prepolarizing field of 6 mT amplitude. Photo depicts the experiment for collection of the spectra in Fig. 6. (B) Same apparatus set up to measure a single-layer pouch cell of outer dimensions 23×12 cm^2, with an active area 5×15 cm^2 filled with 3 mL of the same electrolyte. Corresponds to data shown in panels C and D. For these experiments, the prepolarizing field was directed along the y-direction. (C) Power-spectral-density (PSD) plot showing noise floor of the SQUID gradiometer in the absence of a sample (gray trace), with one single-layer battery (blue trace, panel B), and with a vertical stack of four identical single-layer batteries (pink trace). Data correspond to 5 s of acquisition with 10 kHz sampling rate at a background field of $38.6\,\mu$T. Introduction of the battery under the SQUID dewar increases the measured noise floor from below $300\,\text{aT}/\sqrt{\text{Hz}}$ (in the region of flat response above 400 Hz) by a factor of 100 to around $30\,\text{fT}/\sqrt{\text{Hz}}$—this behavior is expected due to Johnson noise of slabs of electrically conductive materials including the Al pouch-cell foil, which generate thermal magnetic noise (12). The noise floor in the presence of the four-battery stack exhibits a reduction at higher frequencies which may be attributable to the skin effect, warranting further study. (D) Battery-NMR spectra (FFTs) showing the proton signal from the electrolyte in one battery (blue trace) and the four-battery stack (pink trace) at 1644 Hz under application of a $38.6\,\mu$T detection field. To confirm that the observed peak is really a proton signal, the detection field was then increased to $38.9\,\mu$T, which corresponded to a shift of the peak to 1655 Hz, as expected. Due to the relatively low prepolarizing field and the fact that each cell contained only a thin (sub-mm) layer of electrolyte, spectra were collected as the average of 18 000 scans. Given that signal amplitudes scale with spin polarization (Eq. [6]), increasing the prepolarizing field in future studies will allow direct detection of the electrolyte salt from large-format pouch cells.

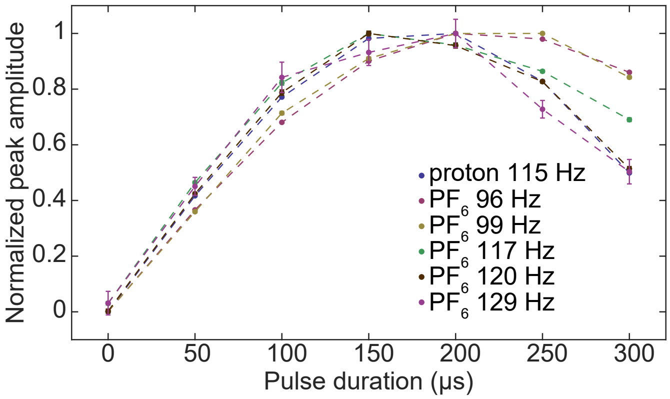

Fig. S12. Nutation curves showing behavior of the solvent-proton and PF_6^- peaks (Fig. S6A) as a function of y-pulse duration in the OPM-based apparatus. The 30 μT DC pulse was applied after shuttling to rotate sample magnetization into the detection plane, as shown in Fig. 1. Although the nutation frequencies of the different peaks are not identical, a 200 μs pulse duration was identified as appropriate for both the proton and PF_6^- spin systems and was therefore used to collect all experimental spectra. The PF_6^- manifold at 87 Hz is not included in this graphic, due to its complex splitting pattern at the 2.7 μT background field (Fig. S6A). Error bars correspond to the standard deviation of noise in the spectral region 132–140 Hz; dashed lines connecting data points are a guide to the eye.

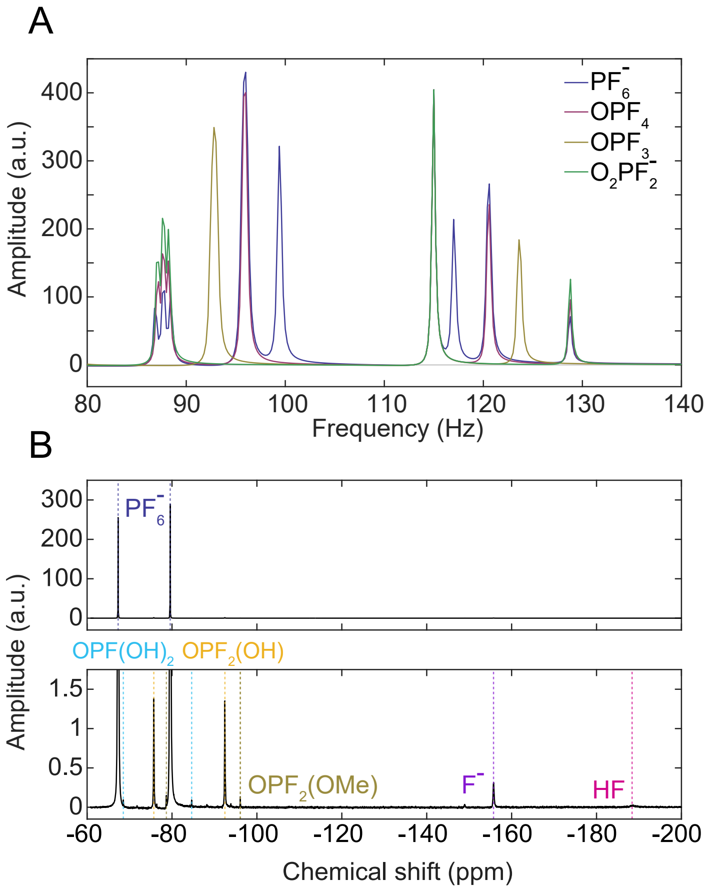

Fig. S13. (A) Ultralow-field simulation of the nZF spectral manifold (cf. Fig. S3) for expected degradation products arising from PF_6^- in the presence of water and metallic ions at a 2.7 μT background field; the peak at 115 Hz is due to ^{1}H. The J-coupling values have been adjusted according to the molecular species using experimental values from (13). Each PF_n spin system with $n = \{2, 3, 4, 6\}$ gives rise to a different spectral pattern, making possible the detection of individual degradation products. (B) High-field experimental spectrum of the PF_6^- manifold in a nominally 2 M 1.5 mL solution of $LiPF_6$ in 50:50 (v/v) EC/DMC (corresponds to the calibration sample 3 in Fig. 7), collected using a 1.4 T benchtop spectrometer. Two PF_6 peaks are visible at chemical shifts of -67 and -80 ppm. Zooming in on the spectrum by a factor of 200 (bottom panel), several degradation products can be observed which are assigned according to literature (10, 14). The sample was measured two weeks after preparation, having been stored in a screwtop vial inside a glovebox. The low amplitude of degradation peaks compared to the PF_6^- peaks suggests that active aging of the electrolyte will be required in future studies to generate degradation products large enough for observation with ZULF-NMR spectrometers.

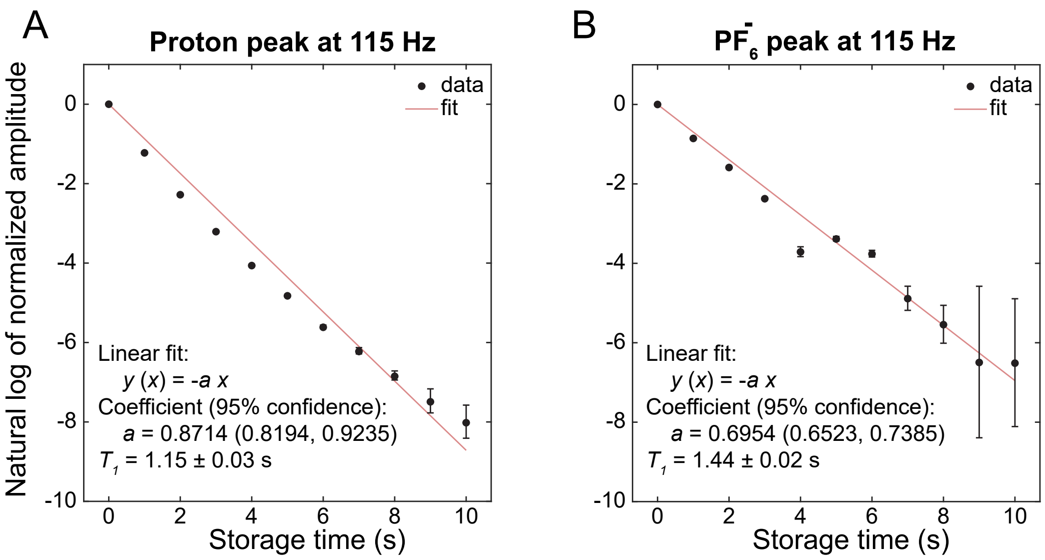

Fig. S14. Relaxation investigation of (A) solvent and (B) solute magnetization using a freshly prepared electrolyte calibration sample (2 M LiPF6 in 50:50 EC/DMC) in the shuttling setup—conducted by varying the storage time in the solenoid, after shuttling and prior to pulse application (see Fig. 1). The measured peak amplitude decays exponentially from its maximum value at zero storage time, with a time constant of T_1. Taking the inverse slope of the fit, we find T_1 times of 1.15 ± 0.03 s for the solvent proton peak and 1.44 ± 0.02 s for the largest PF_6^- peak. Polarization and acquisition times were both 5 s; 256 scans were averaged for each experiment. Error bars on the data points correspond to the standard deviation of noise in the spectral region 132–140 Hz.

References

1. JW Blanchard, D Budker, Zero- to ultralow-field NMR. *eMagRes* **5**, 1395–1410 (2016).
2. Q Stern, K Sheberstov, Simulation of NMR spectra at zero and ultralow fields from A to Z – a tribute to Prof. Konstantin L'vovich Ivanov. *Magn. Reson.* **4**, 87–109 (2023).
3. S Appelt, et al., Paths from weak to strong coupling in NMR. *Phys. Rev. A* **81**, 023420 (2010).
4. H Hogben, M Krzystyniak, G Charnock, P Hore, I Kuprov, Spinach – A software library for simulation of spin dynamics in large spin systems. *J. Magn. Reson.* **208**, 179–194 (2011).
5. A Abragam, *Principles of Nuclear Magnetism (The International Series of Monographs on Physics)*. (Clarendon Press), (1983).
6. DA Barskiy, A Pravdivtsev, Magnetization and polarization of coupled nuclear spins ensembles at high magnetic fields. *ChemPhysChem* p. e202500092 (2025).
7. AM Fabricant, P Put, DA Barskiy, Proton relaxometry of tree leaves at hypogeomagnetic fields. *Front. Plant Sci.* **15**, 1352282 (2024).
8. A Caciagli, RJ Baars, AP Philipse, BW Kuipers, Exact expression for the magnetic field of a finite cylinder with arbitrary uniform magnetization. *J. Magn. Magn. Mater.* **456**, 423–432 (2018).
9. JW Blanchard, D Budker, A Trabesinger, Lower than low: Perspectives on zero- to ultralow-field nuclear magnetic resonance. *J. Magn. Reson.* **323**, 106886 (2021).
10. S Wiemers-Meyer, M Winter, S Nowak, Mechanistic insights into lithium ion battery electrolyte degradation – a quantitative NMR study. *Phys. Chem. Chem. Phys.* **18**, 26595–26601 (2016).
11. JH Storm, P Hömmen, D Drung, R Körber, An ultra-sensitive and wideband magnetometer based on a superconducting quantum interference device. *Appl. Phys. Lett.* **110**, 72603 (2017).
12. J Nenonen, J Montonen, T Katila, Thermal noise in biomagnetic measurements. *Rev. Sci. Instruments* **67**, 2397–2405 (1996).
13. R Fernández-Galán, BR Manzano, A Otero, M Lanfranchi, MA Pellinghelli, ^{19}F and ^{31}P NMR evidence for silver hexafluorophosphate hydrolysis in solution. New palladium difluorophosphate complexes and x-ray structure determination of [Pd(η^3-2-Me-C3H4)(PO2F2)(PCy3)]. *Inorg. Chem.* **33**, 2309–2312 (1994).
14. BS Parimalam, AD MacIntosh, R Kadam, BL Lucht, Decomposition reactions of anode solid electrolyte interphase (SEI) components with $LiPF_6$. *The J. Phys. Chem. C* **121**, 22733–22738 (2017).
